# Targeting ATAD3A-PINK1-mitophagy axis overcomes chemoimmunotherapy resistance by redirecting PD-L1 to mitochondria

**DOI:** 10.1038/s41422-022-00766-z

**Published:** 2023-01-10

**Authors:** Xiao-Qing Xie, Yi Yang, Qiang Wang, Hao-Fei Liu, Xuan-Yu Fang, Cheng-Long Li, Yi-Zhou Jiang, Shuai Wang, Hong-Yu Zhao, Jing-Ya Miao, Shuai-Shuai Ding, Xin-Dong Liu, Xiao-Hong Yao, Wen-Tao Yang, Jun Jiang, Zhi-Ming Shao, Guoxiang Jin, Xiu-Wu Bian

**Affiliations:** 1 Institute of Pathology and Southwest Cancer Center, Southwest Hospital, Third Military Medical University (Army Medical University) and Key Laboratory of Tumor Immunopathology, Ministry of Education of China, Chongqing, China; 2 Department of Oncology, Shandong Second Provincial General Hospital, Jinan, Shandong China; 3 Department of Breast Surgery, Fudan University Shanghai Cancer Center; Key Laboratory of Breast Cancer in Shanghai, Shanghai, China; 4 National Laboratory of Biomacromolecules, Chinese Academy of Sciences Center for Excellence in Biomacromolecules, Institute of Biophysics, Chinese Academy of Sciences, Beijing, China; 5 College of Life Sciences, University of Chinese Academy of Sciences, Beijing, China; 6 Department of Pathology, Fudan University Shanghai Cancer Center, Shanghai, China; 7 Department of Breast Diseases, Southwest Hospital, Third Military Medical University (Army Medical University), Chongqing, China

**Keywords:** Breast cancer, Mitophagy, Cancer immunotherapy

## Abstract

Only a small proportion of patients with triple-negative breast cancer benefit from immune checkpoint inhibitor (ICI) targeting PD-1/PD-L1 signaling in combination with chemotherapy. Here, we discovered that therapeutic response to ICI plus paclitaxel was associated with subcellular redistribution of PD-L1. In our immunotherapy cohort of ICI in combination with nab-paclitaxel, tumor samples from responders showed significant distribution of PD-L1 at mitochondria, while non-responders showed increased accumulation of PD-L1 on tumor cell membrane instead of mitochondria. Our results also revealed that the distribution pattern of PD-L1 was regulated by an ATAD3A-PINK1 axis. Mechanistically, PINK1 recruited PD-L1 to mitochondria for degradation via a mitophagy pathway. Importantly, paclitaxel increased ATAD3A expression to disrupt proteostasis of PD-L1 by restraining PINK1-dependent mitophagy. Clinically, patients with tumors exhibiting high expression of ATAD3A detected before the treatment with ICI in combination with paclitaxel had markedly shorter progression-free survival compared with those with ATAD3A-low tumors. Preclinical results further demonstrated that targeting ATAD3A reset a favorable antitumor immune microenvironment and increased the efficacy of combination therapy of ICI plus paclitaxel. In summary, our results indicate that ATAD3A serves not only as a resistant factor for the combination therapy of ICI plus paclitaxel through preventing PD-L1 mitochondrial distribution, but also as a promising target for increasing the therapeutic responses to chemoimmunotherapy.

## Introduction

Triple-negative breast cancer (TNBC) represents approximately 15%–20% of all primary breast cancers with only a 13- to 18-month median overall survival after metastasis.^1–3^ TNBC treatment remains a great challenge due to lack of specific molecular targets.^4^ Current standard therapeutic strategy for TNBC is utilizing the chemo-drugs such as paclitaxel. Compared with patients with other breast cancer subtypes, TNBC patients have higher PD-L1 expression and more tumor-infiltrating lymphocytes (TILs).^5^ Therefore, with the success of immune checkpoint inhibitor (ICI) therapy in various malignancies,^6–8^ PD-L1-targeting strategy has been investigated in patients with TNBC. However, the efficacy of monotherapy is less than 25% even in patients with PD-L1-positive tumors.^9–12^ In addition, although the chemo-drug nab-paclitaxel has been utilized to combine with anti-PD-L1 antibody atezolizumab to enhance the efficacy of TNBC therapy, the combination therapy only results in a modest improvement in progression-free survival (PFS) of patients, while there is no statistical significance in improvement of overall survival (OS) in IMpassion130 clinical trial.^1^ Another phase 3 IMpassion131 trial of combining paclitaxel with atezolizumab is more disappointing as it failed to show benefit at its primary endpoint of PFS in PD-L1-positive TNBC population,^12,13^ highlighting the need for better understanding of the mechanisms underlying the resistance to anti-PD-1/PD-L1 antibodies in combination with paclitaxel chemotherapy.

PD-L1 protein expressed by tumor cells engages with PD-1 on T cells to escape from antitumor immunity. Targeting the regulation mechanisms of PD-L1 may provide multiple strategies for overcoming the immunosuppression, including transcriptional, translational or posttranslational downregulation of PD-L1.^14^ Interestingly, in addition to the modulation of protein levels, the redistribution of intracellular PD-L1 to cell surface has been found to be associated with reduced T cell-mediated killing.^15–17^ These results call for further investigation of the subcellular redistribution pattern and underlying mechanisms of PD-L1 for developing effective immunotherapy.

Mitochondria participate in multiple cellular biological processes. In addition to their well-known roles as the power houses and metabolic centers, mitochondria are also appreciated for their capacity to regulate the responses of the immune system.^18^ Mitochondria-mediated cellular events include the production of reactive oxygen species (ROS), oxidative phosphorylation, metabolic reprogramming and are involved in the development, activation and function of immune cells.^19–22^ Mitochondria also act as signaling organelles, contributing to the localization of immune regulatory proteins on outer membrane in innate immune responses.^23,24^ However, it is unclear whether mitochondria participate in PD-L1 distribution and expression regulation, and are important for the efficacy of PD-1/PD-L1 targeted therapy.

In this study, we characterized the distribution of PD-L1 at mitochondria and its association with ICI effect. In an immunotherapy cohort, patients with the MITO signature (PD-L1 localized to mitochondria) tumors responded more effectively compared with those with the CM signature (PD-L1 accumulated on cell membrane). Moreover, we revealed that the mitochondrial distribution of PD-L1 is regulated by an ATAD3A-PINK1-mitophagy axis. Paclitaxel upregulates ATAD3A to suppress mitophagy-mediated recruitment and subsequent degradation of PD-L1 protein, resulting in its excessive accumulation on cell membrane to markedly enhance tumor-induced immune deficiency. Therefore, inhibition of ATAD3A may restore a favorable antitumor immunity and improve the therapeutic efficacy of ICI plus paclitaxel for TNBC.

## Results

### Mitochondrial distribution of PD-L1 in TNBC is associated with therapeutic response to ICI plus paclitaxel

**Fig. 1:**
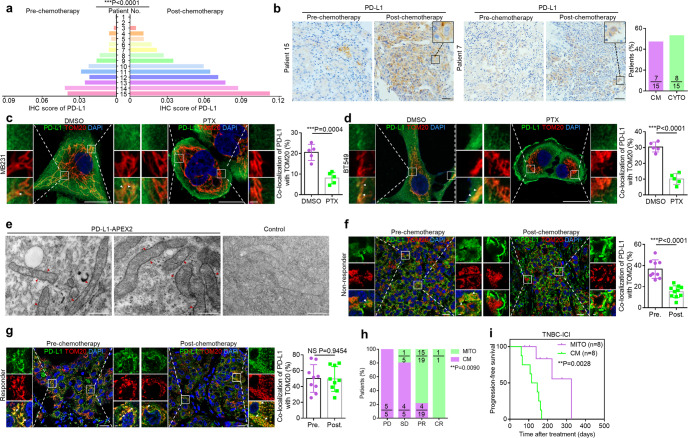
Mitochondrial distribution of PD-L1 is associated with therapeutic response to ICI. **a** Quantitative analysis of pre- and post-chemotherapy IHC score of PD-L1 in human TNBC. IHC score of PD-L1 was determined by IOD value/area. Biopsies were taken as pre-chemotherapy samples at their first to doctor and intraoperative specimens were taken as post-chemotherapy samples after neoadjuvant treatment. Neoadjuvant chemotherapy included TE regimen (paclitaxel/docetaxel, epirubicin) or TEC regimen (docetaxel, epirubicin and cyclophosphamide) (*n* = 15, paired two-sided *t*-test). **b** Left, representative IHC staining for different subcellular patterns of PD-L1 in TNBC samples pre- or post-chemotherapy. Patient 15 represented the cases with increased cell membranal localization of PD-L1 (the CM pattern). Patient 7 represented the cases with cytoplasmic localization of PD-L1 (the CYTO pattern). Right, the percentage of patients with different PD-L1 subcellular distribution. Scale bars, 50 μm. **c**, **d** Left, co-localization of PD-L1 (green) and TOM20-labeled mitochondria (red) with or without 20 nM paclitaxel treatment for 12 h in MDA-MB-231 (**c**) and BT549 cells (**d**). Arrowheads, co-localization. Scale bars, 20 μm and 2 μm (inset). Right, the percentage of PD-L1 co-localized with TOM20 (*n* = 5 fields, *t*-test). **e** EM images of BT549 cells stably expressing PD-L1-APEX2 or control cells. Arrowheads, positive signals of PD-L1-APEX2 on outer mitochondrial membrane. Scale bars, 500 nm. **f**, **g** Left, co-localization of PD-L1 (green) and TOM20-labeled mitochondria (red) in TNBC specimens from a non-responder (**f**) or a responder (**g**) pre- or post-chemotherapy in the immunotherapy cohort from arm C of the FUTURE trial. These patients with refractory metastatic TNBC had received standard chemotherapy including taxol for previous antitumor regimens and underwent anti-PD-1 antibodies plus nab-paclitaxel. Intraoperative specimens and progressive disease biopsies were taken as pre-chemotherapy samples and post-chemotherapy samples respectively. Arrowheads, examples of co-localization. Scale bars, 20 μm and 2 μm (inset). Right, the percentage of PD-L1 co-localized with TOM20 (*n* = 9 fields, *t*-test). **h** The percentages of patients with CM or MITO signature in four different groups on the basis of their best responses to anti-PD-1 plus nab-paclitaxel combination therapy. PD, progressive disease; SD, stable disease; PR, partial response; CR, complete response (*n* = 30, Fisher’s exact test). **i** Kaplan-Meier analysis of the progression-free survival of TNBC patients with CM or MITO signature who received combination therapy (*n* = 16). Data were derived from the immunotherapy cohort of this study (Kaplan-Meier method and log-rank test). Data are representative of at least two independent experiments and are shown as means ± SD. See also Supplementary information, Fig. S1.

Several previous studies suggested that different approaches including some chemotherapeutic agents may improve ICI therapy by enhancing PD-L1 expression. However, the majority of patients remained unresponsive to immunotherapy even with elevated PD-L1 levels.^9,25,26^ For a cohort of patients with TNBC receiving neoadjuvant chemotherapy including taxol, biopsies were taken as pre-chemotherapy samples at their first to doctor and intraoperative specimens were taken as post-chemotherapy samples after neoadjuvant treatment. We found that the immunohistochemistry (IHC) scores of PD-L1 were increased with various degrees after receiving neoadjuvant chemotherapy including taxol (Fig. 1a). Interestingly, in addition to protein level changes, there was an alteration in subcellular distribution of PD-L1 following chemotherapy. Some samples showed decreased cytoplasm localization of PD-L1 accompanied by increased localization on cell membrane after chemotherapy, while others exhibited marked cytoplasm localization of PD-L1 (Fig. 1b). These results indicate that chemotherapy including taxol not only interferes with PD-L1 expression but also redistributes PD-L1 with potential implication for therapy efficacy.

Taxol, a standard treatment for TNBC, is the first chemo-drug approved by FDA to combine with anti-PD-1/PD-L1 antibodies. To investigate the mechanisms underlying PD-L1 redistribution upon taxol chemotherapy in TNBC, we treated TNBC cells with paclitaxel and found that the localization of PD-L1 on endoplasmic reticulum or Golgi complex showed no significant difference compared with control cells (Supplementary information, Fig. S1a, b). However, a significant amount of PD-L1 co-localized with TOM20-labeled mitochondria, and the co-localization was reduced in paclitaxel-treated TNBC cells. With decreased mitochondrial localization, PD-L1 markedly accumulated on cell surface (Fig. 1c, d; Supplementary information, Fig. S1c and Videos S1–S4). APEX2-labeled specific protein imaging by electron microscopy (EM) revealed that PD-L1–APEX2 complex was enriched on the mitochondrial outer membrane (Fig. 1e), suggesting that PD-L1 may localize at mitochondria and the process is prevented by paclitaxel.

To determine whether mitochondrial distribution of PD-L1 was associated with therapeutic response to ICI, we detected patients with refractory metastatic TNBC who had received previous antitumor regimens including taxol and underwent treatment with anti-PD-1 antibodies plus nab-paclitaxel in the FUTURE trial.^27^ Intraoperative specimens were taken as pre-chemotherapy samples, while progressive disease biopsies before chemoimmunotherapy were taken as post-chemotherapy samples. Immunofluorescent staining showed that tumor samples from non-responders to ICI plus nab-paclitaxel exhibited less mitochondrial PD-L1 but more PD-L1 accumulation on cell membrane especially after adjuvant chemotherapy including taxol, referred to as CM signature (Fig. 1f). In contrast, the samples from responders exhibited significant mitochondrial localization of PD-L1 both in pre- and post- chemotherapy lesions, referred to as MITO signature (Fig. 1g). Our finding further revealed that the TNBC patients with MITO signature displayed better therapeutic responses to ICI plus nab-paclitaxel treatment (partial response or complete response) compared with those with CM signature (Fig. 1h). In addition, Kaplan-Meier survival analysis indicated that the TNBC patients with CM signature at baseline (before being treated with anti-PD-1 antibodies plus nab-paclitaxel) had markedly shorter PFS compared with those with MITO signature (Fig. 1i). Taken together, our study demonstrates the taxol-induced changes of subcellular PD-L1 redistribution, which are correlated with the therapeutic responses to ICI plus nab-paclitaxel. Moreover, we showed that mitochondrial distribution of PD-L1 contributes to the efficacy of the combination therapy.

### ATAD3A prevents PD-L1 distribution to mitochondria and its level correlates with ICI resistance

**Fig. 2:**
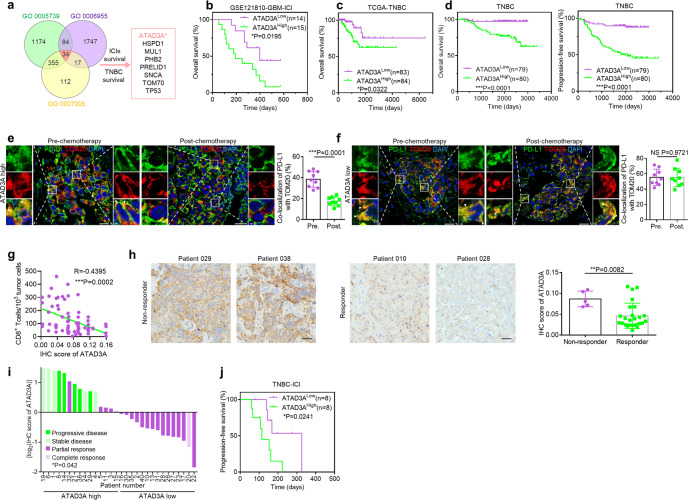
ATAD3A prevents PD-L1 distribution to mitochondria. **a** Venn diagram depicting the overlapped common genes in the mitochondrion component set (GOCC 0005739), mitochondrion organization set (GOBP 0007005), and immune response set (GOBP 0006955). **b** Kaplan-Meier analysis of the OS of glioblastoma patients who received anti-PD-1 antibody treatment with high or low ATAD3A mRNA expression (*n* = 29). Data were derived from GSE121810 cohort. Cutoff of ATAD3A expression was determined by the median (Kaplan-Meier method and log-rank test). **c** Kaplan-Meier analysis of the OS of TNBC patients with high or low ATAD3A transcriptional level. Data were derived from TCGA database. Cutoff of ATAD3A expression was determined by the median (Kaplan-Meier method and log-rank test). **d** Kaplan-Meier analysis of the OS (left) and PFS (right) of TNBC patients with high or low ATAD3A expression from the multicentric clinical cohort (Kaplan-Meier method and log-rank test). These patients underwent radical mastectomy or modified mastectomy and received the standard chemotherapy. **e**, **f** Left, co-localization of PD-L1 (green) and TOM20-labeled mitochondria (red) in TNBC specimens with high (**e**) or low (**f**) ATAD3A expression pre- or post-chemotherapy including taxol in immunotherapy cohort. Arrowheads, co-localization. Scale bars, 20 μm and 2 μm (inset). Right, the percentage of PD-L1 co-localized with TOM20 (*n* = 9 fields, *t*-test). **g** The correlation between ATAD3A levels and the number of tumor-infiltrating CD8^+^ T cells in TNBC tumors (*n* = 67). IHC score was quantified by Image-Pro Plus software 6.0, three to five fields per section (Pearson correlation analysis). **h** Representative images (left) and IHC score (right) of ATAD3A expression in tumors from non-responders and responders before anti-PD-1 antibody plus nab-paclitaxel therapy in immunotherapy cohort. Scale bars, 50 μm (*n* = 30, Mann-Whitney U test). **i** Patients with different responses in the immunotherapy cohort. Subjects were colored by their responses to combination therapy. CR, complete response; PR, partial response; SD, stable disease; PD, progressive disease. *y*-axis was determined by IHC score of ATAD3A by logarithm to the base 2. Cutoff of ATAD3A expression was determined by the median (*n* = 30, Fisher’s exact test). **j** Kaplan-Meier analysis of the progression-free survival of TNBC patients with high or low ATAD3A expression receiving combination therapy (*n* = 16). Data are derived from the immunotherapy cohort (Kaplan-Meier method and log-rank test). See also Supplementary information, Fig. S2.

Given that mitochondrial distribution of PD-L1 in TNBC is linked to beneficial outcome of ICI therapy, we explored the factors contributing to PD-L1 mitochondrial localization with three gene sets including mitochondrion component set (GOCC 0005739), mitochondrion organization set (GOBP 0007005) and immune response set (GOBP 0006955) and found 34 related genes. We also performed analysis of these 34 survival-related genes in anti-PD-1-treatment cohort (GSE121810) and TNBC cohort from The Cancer Genome Atlas (TCGA) (Fig. 2a). Data from anti-PD-1 glioblastoma cohort (GSE121810) indicate that 8 among 34 genes were strongly associated with the survival of patients with PD-1-targeting therapy (Fig. 2b; Supplementary information, Fig. S2a). We then linked 8 candidate genes to patient survival in TNBC cohort from TCGA and found only one gene, *ATPase family AAA domain-containing 3 A* (*ATAD3A*) was related to the survival rate of patients (Fig. 2c; Supplementary information, Fig. S2b). This gene is a member of the ATPase family AAA-domain containing 3 gene family and mainly encodes mitochondrial protein participating in multiple biological processes, which has been observed to promote tumor progression by enhancing the ability of tumor migration and chemotherapy resistance.^28–30^ In fact, ATAD3A is an effective predictor of clinical outcomes of patients with TNBC (Fig. 2c; Supplementary information, Fig. S2c). We then examined the expression of ATAD3A in 399 breast cancer samples from the multicentric clinical cohort where patients underwent mastectomy and received the standard chemotherapy. Kaplan-Meier analysis revealed that high expression of ATAD3A was significantly associated with shortened PFS and OS of patients with TNBC but not those with other breast cancer subtypes. (Fig. 2d; Supplementary information, Fig. S2d–l).

Importantly, in our immunotherapy cohort, patients with ATAD3A-high tumors showed the CM distribution signature of PD-L1, while others with ATAD3A-low tumors exhibited MITO signature after chemotherapy including taxol (Fig. 2e, f). In addition, IHC staining revealed that the expression level of ATAD3A was negatively correlated with the number of infiltrating CD8^+^ T cells (Fig. 2g). To gain more insights into the clinical significance of ATAD3A in predicting patient responses to immunotherapy, we evaluated the correlation between the protein levels of ATAD3A and therapeutic responses. ATAD3A expression was considerably higher in tumors from non-responders than that in tumors from responders (Fig. 2h). Patients with ATAD3A-high tumors showed stable or progressive diseases after combination therapy, while patients with ATAD3A-low tumors responded well to the therapy (Fig. 2i). Moreover, high expression of ATAD3A protein was associated with poor survival of patients with TNBC who received treatment with anti-PD-1 antibody plus nab-paclitaxel (Fig. 2j). These findings indicate that both ATAD3A levels and PD-L1 redistribution are correlated with the therapeutic responses to ICI plus paclitaxel.

### ATAD3A-PINK1 mitophagy axis is involved in the redistribution and degradation of PD-L1

**Fig. 3:**
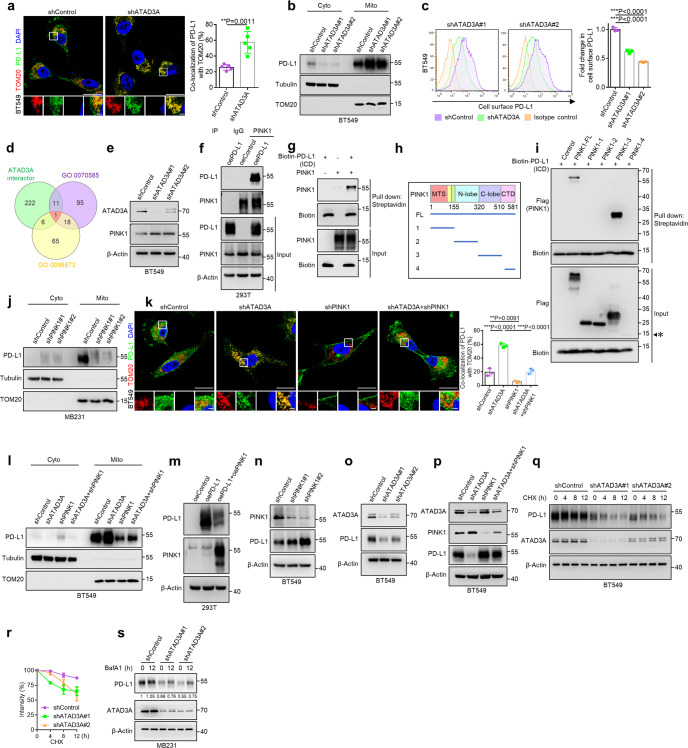
Involvement of ATAD3A-PINK1 mitophagy in the distribution and degradation of PD-L1. **a** Left, immunostaining of PD-L1 (green) and TOM20-labeled mitochondria (red) in BT549 human TNBC cells with or without ATAD3A-knockdown (shATAD3A#1 and shATAD3A#2). Scale bars, 20 μm and 2 μm (inset). Right, the percentage of PD-L1 co-localized with TOM20 (*n* = 5 fields, *t*-test). **b** Immunoblot of PD-L1 in the cytoplasm and mitochondria of control and ATAD3A-knockdown BT549 cells. TOM20 and Tubulin were used as mitochondria and cytoplasm protein controls, respectively. Cyto, cytoplasm; mito, mitochondria. **c** Flow cytometry (left) and quantification (right) of surface PD-L1 in control and ATAD3A-knockdown BT549 cells (*n* = 3, one-way ANOVA). **d** Venn diagram depicting overlapped genes for the interaction protein of ATAD3A set (BioGRID, RP5-832C2.1), the protein localization to mitochondrion set (GOBP 0070585) and the intrinsic component of mitochondrial membrane (GOCC 0098573). **e** Immunoblot of PINK1 in control and ATAD3A-knockdown BT549 cells. **f** Immunoblot of PD-L1 and PINK1 in HEK293T cells overexpressing PD-L1 (OE-PD-L1) and control cells (OE-Control), assessed after immunoprecipitation with immunoglobulin G (IgG) or antibody to PINK1. **g** Protein direct interaction analysis of the intracellular domain of PD-L1 (ICD) and PINK1 in vitro. Purified Flag-labeled full-length PINK1 was incubated with Biotin-labeled PD-L1 ICD domain, followed by streptavidin pull-down and immunoblot. **h** Schematic diagram of Flag-labeled full-length (FL) and truncated mutants with indicated domains (amino acids 1–155, amino acids 156–320, amino acids 321–509, amino acids 510–581) of PINK1. MTS, mitochondrial targeting sequence; N-lobe, kinase domain N; C-lobe, kinase domain C; CTD, C-terminal domain. **i** Protein direct interaction analysis of the intracellular domain of PD-L1 (ICD) and truncated PINK1 mutants in vitro. Purified Flag-labeled full-length and truncated PINK1 were incubated with Biotin-labeled PD-L1 ICD domain, followed by streptavidin pull-down and immunoblot. The estimated size of PINK1-4 (amino acids 510–581) which did not express in HEK293T cells was labeled with asterisk. **j** Immunoblot of PD-L1 in the cytoplasm and mitochondria of MDA-MB-231 cells with or without PINK1-knockdown (shPINK1#1 and shPINK1#2). TOM20 and Tubulin were used as mitochondria and cytoplasm protein controls. Cyto, cytoplasm; mito, mitochondria. **k** Left, co-localization of PD-L1 (green) and TOM20 (red) in control, ATAD3A knockdown, PINK1 knockdown or ATAD3A and PINK1 double knockdown BT549 cells. Scale bars, 20 μm and 2 μm (inset). Right, the percentage of PD-L1 co-localized with TOM20 (*n* = 5 fields, one-way ANOVA). **l** Immunoblot of PD-L1 in the cytoplasm and mitochondria of control, ATAD3A-knockdown, PINK1-knockdown or ATAD3A and PINK1 double knockdown BT549 cells. **m** Immunoblot of indicated proteins in PD-L1-transfected HEK293T cells with or without PINK1 overexpression. **n** Immunoblot of PD-L1 in control and PINK1-knockdown (shPINK1#1 and shPINK1#2) BT549 cells. **o** Immunoblot of PD-L1 in BT549 cells transfected with control shRNA or shATAD3A (shATAD3A#1 and shATAD3A#2). **p** Immunoblot of total PD-L1 in control, ATAD3A-knockdown, PINK1-knockdown or ATAD3A and PINK1 double knockdown BT549 cells. **q** Immunoblot of PD-L1 in control and ATAD3A-knockdown BT549 cells treated with 20 μM CHX for indicated times. h, hours. **r** Quantification of PD-L1 intensity in immunoblot in control and ATAD3A-knockdown BT549 cells. **s** Immunoblot of PD-L1 in control and ATAD3A-knockdown MDA-MB-231 cells incubated with 20 nM BafA1 for indicated times. h, hours. Data are representative of at least two independent experiments and are shown as means ± SD. See also Supplementary information, Figs. S3 and S4.

To investigate how ATAD3A regulates the redistribution of PD-L1, we knocked down ATAD3A with shRNAs in TNBC cells. Immunofluorescence staining showed that PD-L1 more frequently co-localized with TOM20 at mitochondria in ATAD3A-knockdown TNBC cells (Fig. 3a; Supplementary information, Fig. S3a). However, reintroduction of ATAD3A in its knockdown cells reduced the mitochondrial distribution of PD-L1 (Supplementary information, Fig. S3b, c). Analysis of isolated mitochondria also indicated that ATAD3A-knockdown enhanced PD-L1 translocation from cytoplasm into mitochondria (Fig. 3b; Supplementary information, Fig. S3d, e). With the enhancement of mitochondrial PD-L1, cell surface PD-L1 was decreased with ATAD3A disruption (Fig. 3c; Supplementary information, Fig. S3f).

To further understand whether any proteins function downstream of ATAD3A to regulate PD-L1 mitochondrial localization, we analyzed three protein or gene sets including interaction protein of ATAD3A set (BioGRID, RP5-832C2.1), protein localization to mitochondrion set (GOBP 0070585) and intrinsic component of mitochondrial membrane set (GOCC 0098573). PTEN-induced kinase 1 (PINK1) was identified in the overlap of datasets that functions as a key mediator of mitophagy (Fig. 3d). The accumulation of PINK1 on the outer mitochondrial membrane (OMM) activates mitophagy by facilitating Parkin translocation to the mitochondrial surface.^31–34^ Our previous study has demonstrated that ATAD3A negatively regulates PINK1 accumulation by promoting its transport from OMM to the inner mitochondrial membrane (IMM), where PINK1 protein undergoes cleavage.^35^ We then assessed protein level of PINK1 in ATAD3A-knockdown TNBC cells and found increased level of PINK1 compared with control cells (Fig. 3e; Supplementary information, Fig. S3g). To determine whether increased PINK1 was required for recruiting PD-L1 to mitochondria, we performed co-immunoprecipitation (Co-IP) assay and found that PINK1 interacted with PD-L1 in HEK293T cells transfected to express the full length (FL) of PD-L1 rather than the extracellular domain (ECD) of PD-L1 (Fig. 3f; Supplementary information, Fig. S3h, i). Moreover, PINK1 was found to directly interact with the intracellular domain of PD-L1 (Fig. 3g; Supplementary information, Fig. S3j). To identify the binding region of PINK1 with PD-L1, we generated orderly truncated PINK1 proteins from HEK293T cells and found that the C-lobe of kinase domain of PINK1 was required for interaction with the intracellular region of PD-L1 (Fig. 3h, i). Furthermore, we generated PINK1-knockdown cells and found that PD-L1 co-localized with TOM20-labeled mitochondria in control, but the co-localization was significantly decreased in PINK1-knockdown cells (Supplementary information, Fig. S3k, m). However, reintroduction of PINK1 in its knockdown cells increased mitochondrial localization of PD-L1 (Supplementary information, Fig. S3l, n). Western blot assay revealed that the mitochondrial distribution of PD-L1 was abrogated upon PINK1 depletion (Fig. 3j; Supplementary information, Fig. S3o). In addition, we utilized mitochondria damaging agent carbonyl cyanide-m-chlorophenylhydrazone (CCCP) to activate PINK1 in TNBC cells. Subsequently, we found that PD-L1 mitochondrial localization was increased after CCCP treatment (Supplementary information, Fig. S3p–r). Taken together, the results indicate that PINK1 acts as a bridge for PD-L1 recruitment to mitochondria.

To investigate the role of ATAD3A-PINK1 axis in regulating PD-L1 redistribution, we generated ATAD3A and PINK1 single or double knockdown TNBC cells. We found that knockdown of PINK1 inhibited the increase of PD-L1 at mitochondria by ATAD3A depletion (Fig. 3k; Supplementary information, Fig. S4a). ATAD3A knockdown caused a decreased level of PD-L1 in the cytoplasmic fractions but an increase in the mitochondrial fractions, in contrast to the effect of PINK1 knockdown (Fig. 3l; Supplementary information, Fig. S4b, c). In addition to recruiting PD-L1 to mitochondria, overexpression of PINK1 was also associated with a decrease in PD-L1 level (Fig. 3m). Interestingly, we further generated PINK1 kinase-dead mutants and found that the mutants also suppressed PD-L1 to the similar level of wild-type PINK1, suggesting that PINK1 regulates PD-L1 independent of its kinase activity (Supplementary information, Fig. S4d). By contrast, PINK1-knockdown resulted in an enhanced level of PD-L1 (Fig. 3n; Supplementary information, Fig. S4e). Consequently, we observed that the total protein of PD-L1, rather than the transcripts, was reduced in ATAD3A-knockdown cells, which was restored by concurrent PINK1 deletion (Fig. 3o, p; Supplementary information, Fig. S4f–i). Given that mitophagy also removes mitochondria-associated protein in addition to damaged mitochondria, we hypothesized that PD-L1 localized at mitochondria might be degraded in a mitophagy-dependent manner, resulting in significant reduction in its level. By using cycloheximide (CHX) to inhibit protein synthesis, we found more rapidly declined PD-L1 level in ATAD3A-knockdown cells in comparison with control cells (Fig. 3q, r; Supplementary information, Fig. S4j, k). Notably, autophagy/mitophagy inhibition with bafilomycin A1 (BafA1) substantially increased PD-L1 level in ATAD3A-knockdown cells than in control cells (Fig. 3s; Supplementary information, Fig. S4l, m). These results indicate that ATAD3A inhibits PINK1 function in TNBC cells, thereby preventing PD-L1 recruitment to mitochondria for mitophagy-mediated degradation.

### Paclitaxel upregulates ATAD3A to suppress PINK1 and mitophagy to promote immunotherapy resistance

**Fig. 4:**
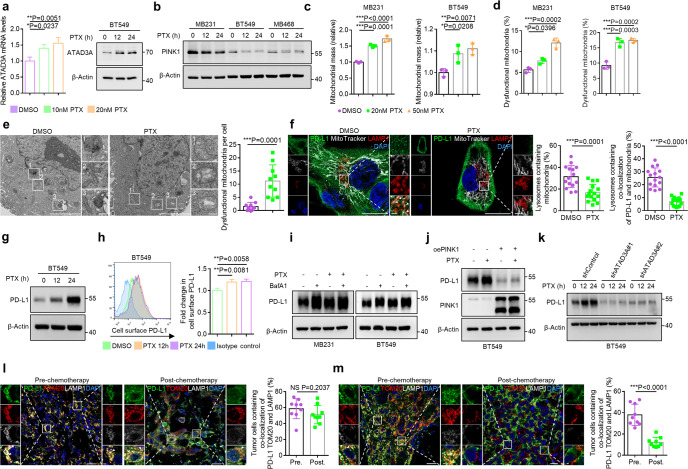
Paclitaxel upregulates ATAD3A to suppress PINK1-dependent mitophagy. **a** Left, qRT-PCR of ATAD3A mRNA in BT549 cells in the presence of paclitaxel (10 nM or 20 nM) for 12 h (*n* = 3, one-way ANOVA). Right, immunoblot of ATAD3A in BT549 cells incubated with 20 nM paclitaxel for 12 h and 24 h. **b** Immunoblot of PINK1 after treatment with 20 nM paclitaxel for 12 h and 24 h in TNBC cell lines. **c**, **d** Quantification of mitochondrial mass (**c**) and the percentage of dysfunctional mitochondria (**d**) in TNBC cells in the presence of paclitaxel (20 nM or 50 nM) for 16 h. DMSO was used as a control (*n* = 3, one-way ANOVA). **e** Left, EM images of mitochondria in BT549 cells with 20 nM paclitaxel or DMSO treatment for 16 h. Scale bars, 5 μm and 500 nm (inset). Right, quantification of the number of dysfunctional mitochondria per cell (*n* = 10, *t*-test). **f** Left, co-localization of PD-L1 (green), mitochondria (white) and LAMP1-labeled lysosomes (red) with or without 20 nM paclitaxel treatment for 12 h in MDA-MB-231 cells. Mitochondria were labeled with MitoTracker Deep Red dye. Arrowheads indicate LAMP1-labeled lysosomes containing co-localization of PD-L1 and mitochondria. Airyscan images were shown. Scale bars, 20 μm and 2 μm (inset). Right, the percentages of lysosomes containing mitochondria or containing co-localization of PD-L1 and mitochondria per field (*n* = 15 fields, *t*-test). **g** Immunoblot of PD-L1 in BT549 cells with 20 nM paclitaxel treatment for indicated times. h, hours. **h** Flow cytometry (left) and quantification (right) of surface PD-L1 in BT549 cells treated with 20 nM paclitaxel for indicated times. h, hours (*n* = 3, one-way ANOVA). **i** Immunoblot of PD-L1 in TNBC cells treated with 20 nM BafA1 alone, 20 nM paclitaxel alone or both for 12 h. **j** Immunoblot of PD-L1 in control and PINK1-overexpressing BT549 cells treated with or without 20 nM paclitaxel for 24 h. **k** Immunoblot of PD-L1 in control and ATAD3A-knockdown BT549 cells treated with 20 nM paclitaxel for indicated times. h, hours. **l**, **m** Left, co-localization of PD-L1 (green), TOM20-labeled mitochondria (red) and LAMP1-labeled lysosomes (white) in tumor specimens from a patient with complete response (**l**) or a patient with progressive disease (**m**) pre- or post-chemotherapy in the immunotherapy cohort. Arrowheads, co-localization. Scale bars, 20 μm and 2 μm (inset). Right, the percentage of tumor cells containing co-localization among PD-L1, TOM20 and LAMP1 per field (*n* = 9 fields, *t*-test). Data are representative of at least two independent experiments and are shown as means ± SD. See also Supplementary information, Figs. S5 and S6.

To explore the capacity of paclitaxel to induce PD-L1 redistribution via the ATAD3A-PINK1 mitophagy pathway, we treated TNBC cells with paclitaxel and found increased mRNA and protein expression of ATAD3A (Fig. 4a; Supplementary information, Fig. S5a). The key mitophagy activator PINK1 was concomitantly reduced after treatment (Fig. 4b). Reduction of PINK1 caused the elevation in total mitochondrial mass as well as dysfunctional mitochondria, suggesting that PINK1-dependent mitophagy could be inhibited (Supplementary information, Fig. S5b). The similar phenotypes were exhibited in TNBC cells with paclitaxel treatment (Fig. 4c, d; Supplementary information, Fig. S5c, d). EM demonstrated that paclitaxel increased the abundance of swelling mitochondria with fractured cristae (Fig. 4e). Moreover, the localization of mitochondria in LAMP1-labeled lysosomes was markedly reduced after paclitaxel treatment (Fig. 4f). These results indicate that paclitaxel suppresses PINK1-dependent mitophagy.

Since paclitaxel upregulated ATAD3A and inhibited the PINK1-mitophagy axis important for maintaining the proteostasis of PD-L1, we further investigated whether abnormal PD-L1 distribution caused by paclitaxel was due to mitophagy dysregulation. We found that the co-localization of PD-L1 with mitochondria and lysosomes was markedly reduced with paclitaxel treatment, suggesting the inhibition of a mitophagy-mediated degradation pathway of PD-L1 (Fig. 4f). In support of this notion, both total cellular and cell surface levels of PD-L1 protein were increased without significant transcriptional changes after paclitaxel treatment (Fig. 4g, h; Supplementary information, Fig. S5e–g). We further utilized paclitaxel and a mitophagy/lysosome inhibitor, BafA1, to treat the cells and found either paclitaxel or BafA1 enhanced the accumulation of PD-L1 in the cells. However, the level of PD-L1 was not further increased in cells treated with both paclitaxel and BafA1 as compared with the cells treated with one agent alone (Fig. 4i). This confirms that the inhibition of lysosome-dependent degradation might account for the accumulation of PD-L1. We further activated mitophagy by overexpressing PINK1 in paclitaxel-treated cells and observed abrogation of aberrant accumulation of PD-L1 (Fig. 4j; Supplementary information, Fig. S5h). Similarly, the paclitaxel-induced degree of increase of PD-L1 level is more in control cells than in ATAD3A-low cells where mitophagy was activated (Fig. 4k; Supplementary information, Fig. S5i, j).

To further determine whether mitophagy-mediated PD-L1 degradation pathway was related to therapeutic response to ICI, we localized PD-L1 in tumor samples from the immunotherapy cohort. The results revealed that tumors from patients with complete or partial response to ICI exhibited increased co-localization of PD-L1 with mitochondria and lysosomes, while tumors from patients with progressive disease showed more PD-L1 redistribution on cell membrane without mitophagy-associated localization especially after chemotherapy including taxol treatment (Fig. 4l, m).

These results suggest that paclitaxel upregulates ATAD3A and reduces PINK1-mediated mitophagy to disrupt PD-L1 proteostasis by blocking an intrinsic degradation pathway and facilitating high level accumulation, resulting in a severely immunosuppressed tumor microenvironment that causes failure of immunotherapy.

Besides paclitaxel, we also used other microtubule inhibitors (colchicine and nocodazole) to treat TNBC cells. Both of colchicine and nocodazole could upregulate the mRNA and protein level of ATAD3A (Supplementary information, Fig. S6a–d). Consequently, we further found that the protein level of PD-L1 was increased in TNBC cells after incubating with microtubule inhibitors. In addition, the mitochondrial localization of PD-L1 was suppressed with colchicine and nocodazole treatment (Supplementary information, Fig. S6e, f), suggesting that other microtubule inhibitors might also upregulate ATAD3A level to control PD-L1 localization.

### ATAD3A inhibition evokes a favorable tumor immune microenvironment

**Fig. 5:**
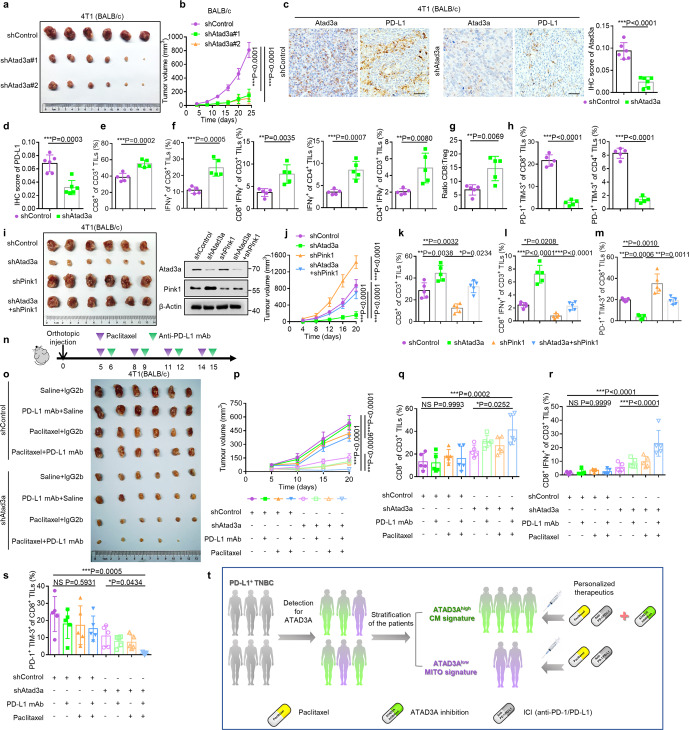
Depletion of Atad3a evokes a favorable tumor immune microenvironment and improves the efficacy of ICI. **a**–**h** BALB/c mice were inoculated orthotopically with 5 × 10^4^ 4T1 cells transfected with control shRNA (shControl) or shRNA for Atad3a (shAtad3a#1 and shAtad3a#2). **a**, **b** The endpoint tumor images (**a**) and volume (**b**) of tumors formed by control and Atad3a-knockdown cells in BALB/c mice (*n* = 6, one-way ANOVA). **c** Left, IHC staining of Atad3a and PD-L1 on serial sections of tumors formed by control and Atad3a-knockdown cells. Scale bars, 50 μm. Right, IHC score of Atad3a in control and Atad3a-knockdown tumors (*n* = 6 fields, *t*-test). **d** IHC score of PD-L1 in control and Atad3a-knockdown tumors (*n* = 6 fields, *t*-test). **e** Quantification of the percentage of tumor-infiltrating CD8^+^ T cells in tumors formed by control and Atad3a-knockdown cells by flow cytometry (*n* = 5, *t*-test). **f** Quantification of the percentages of tumor-infiltrating IFNγ^+^CD8^+^ T cells and IFNγ^+^CD4^+^ T cells by flow cytometry (*n* = 5, *t*-test). **g** Ratio of CD8^+^ cytotoxic T lymphocytes to CD4^+^CD25^+^Foxp3^+^ T_reg_ cells (*n* = 5, *t*-test). **h** Quantification of the percentages of PD-1^+^TIM-3^+^CD8^+^ T cells (left) and PD-1^+^TIM-3^+^CD4^+^ T cells (right) by flow cytometry (*n* = 5, *t*-test). **i**–**m** BALB/c mice were inoculated orthotopically with 5 × 10^4^ 4T1 cells transfected with control shRNA (shControl) or shRNA specific for Atad3a (shAtad3a), Pink1 (shPink1) or both (shAtad3a + shPink1). **i**, Left, the endpoint images of tumors formed by control, Atad3a-knockdown, Pink1-knockdown or Atad3a and Pink1 double knockdown 4T1 cells in BALB/c mice. Right, immunoblot of Atad3a and Pink1 in these 4T1 cells. **j** The volume of tumors mentioned above (*n* = 6, one-way ANOVA). **k** Quantification of the percentage of tumor-infiltrating CD8^+^ T cells by flow cytometry (*n* = 5, one-way ANOVA). **l** Quantification of the percentage of tumor-infiltrating IFNγ^+^CD8^+^ T cells by flow cytometry (*n* = 5, one-way ANOVA). **m** Quantification of the percentage of PD-1^+^TIM-3^+^CD8^+^ T cells by flow cytometry (*n* = 5, one-way ANOVA). **n**–**s** 4T1 tumors formed by control and Atad3a-knockdown cells were established orthotopically in BALB/c mice and received vehicle, anti-PD-L1 antibody (PD-L1 mAb), paclitaxel (PTX) or combined anti-PD-L1 antibody with paclitaxel treatment (PD-L1 mAb + PTX). IgG2b and saline were used as controls. **n** Experimental protocol. **o**, **p** The endpoint tumor images (**o**) and the volume (**p**) of tumors (*n* = 6, one-way ANOVA). **q**–**s** Quantification of the percentages of tumor-infiltrating CD8^+^ T cells (**q**), IFNγ^+^CD8^+^ T cells (**r**) and PD-1^+^TIM-3^+^CD8^+^ T cells (**s**) in 4T1 tumors formed by control and Atad3a-knockdown cells received treatments as described above, determined by flow cytometry (*n* = 5, one-way ANOVA). **t** Schematic model. Patients with PD-L1-positive TNBC could be divided into two groups based on ATAD3A expression. Patients with ATAD3A-high tumors might respond more poorly to ICIs plus paclitaxel therapy, and inhibition of ATAD3A is required to improve clinical outcome. Patients with ATAD3A-low tumors might benefit significantly from ICIs plus paclitaxel combination therapy. See also Supplementary information, Figs. S7–S9.

Since paclitaxel as a standard chemotherapy drug enhances the level of ATAD3A which is highly correlated with poor patient prognosis, we hypothesized that inhibition of ATAD3A may improve the clinical outcome of patients with TNBC. To determine the function of Atad3a in tumor growth, we used RNA interference to deplete Atad3a in 4T1 murine breast cancer cells (Supplementary information, Fig. S7a). Atad3a knockdown did not change the in vitro proliferation of tumor cells or their capacity to form tumors in immunodeficient BALB/c nude mice (Supplementary information, Fig. S7b–d). However, when we inoculated Atad3a-knockdown 4T1 cells into immune-competent BALB/c mice, the growth rate of tumors was reduced in comparison with control cells (Fig. 5a, b; Supplementary information, Fig. S7e, f), suggesting that Atad3a deletion might evoke favorable immune responses to suppress tumor growth. In support of this hypothesis, IHC staining of paraffin tumor sections showed reduction of PD-L1^+^ cells, but increased infiltration of CD8^+^ T cells and granzyme B secretion in Atad3a-deficient tumors, with no changes in the number of Ki67^+^ tumor cells (Fig. 5c, d; Supplementary information, Fig. S7g). Flow cytometry revealed that Atad3a-deficiency increased the proportion of infiltrating CD8^+^ T cell populations as well as activated T lymphocytes, as indicated by increased IFNγ^+^CD8^+^ T cells and IFNγ^+^CD4^+^ T cells (Fig. 5e, f; Supplementary information, Fig. S7h). There was also an increased ratio of CD8^+^ T cells to CD4^+^CD25^+^Foxp3^+^ regulatory T cells (T_reg_) (Fig. 5g; Supplementary information, Fig. S7h). In contrast, the proportion of exhausted T cells expressing surface PD-1 and TIM-3 decreased in tumors formed by Atad3a-knockdown cells (Fig. 5h; Supplementary information, Fig. S7h). In addition to increased proportion, the numbers of infiltrating CD3^+^, CD8^+^, CD4^+^ and activated T cells, including IFNγ^+^CD8^+^ and IFNγ^+^CD4^+^ T cells were also increased, whereas the numbers of exhausted T cells, including PD-1^+^TIM-3^+^CD8^+^ and PD-1^+^TIM-3^+^CD4^+^ T cells decreased in tumors formed by Atad3a-knockdown cells. The number of infiltrating T_reg_ cells showed no significant difference between two tumor groups (Supplementary information, Fig. S7i). We found no significant difference in infiltrating macrophages, myeloid-derived suppressor cells or natural killer cells in tumors formed by Atad3a-knockdown or control cells (Supplementary information, Fig. S7j–l).

Further investigations of the role of Atad3a-Pink1-mitophagy axis in regulating in vivo antitumor immunity showed that simultaneous knockdown of Pink1 reversed the effect of Atad3a knockdown on tumor growth (Fig. 5i, j; Supplementary information, Fig. S8a, b). The increased proportion of total CD8^+^, IFNγ^+^CD8^+^ and IFNγ^+^CD4^+^ T cells as well as the ratio of CD8^+^ to T_reg_ cells in tumors formed by Atad3a-knockdown cells were restored in tumors formed by Atad3a and Pink1 double knockdown cells (Fig. 5k, l; Supplementary information, Fig. S8c, d). In addition, the proportion of PD-1^+^TIM-3^+^CD8^+^ T cells and PD-1^+^TIM-3^+^CD4^+^ T cells upon knockdown of Atad3a was rescued to the same level in tumors with further Pink1 knockdown as compared with control tumors (Fig. 5m; Supplementary information, Fig. S8e). In contrast to Atad3a-knockdown-mediated significant increase in activated immune state and decrease in exhausted state, the numbers of various T cell populations including infiltrating CD3^+^, CD8^+^, CD4^+^, IFNγ^+^CD8^+^, IFNγ^+^CD4^+^ and PD-1^+^TIM-3^+^CD8^+^ T cells were restored to the level of control tumors upon simultaneous knockdown of Atad3a and Pink1 with the exception for the numbers of PD-1^+^TIM-3^+^CD4^+^ T cells and T_reg_ cells (Supplementary information, Fig. S8f, g). Therefore, suppression of Atad3a reduces tumor progression by facilitating antitumor immune responses, and the effect was dependent on Pink1, indicating a crucial role of the Atad3a-Pink1 mitophagy axis in the regulation of tumor immune microenvironment.

### Inhibition of ATAD3A improves the efficacy of ICI in combination with paclitaxel

The fact that ATAD3A inhibition restores the beneficial antitumor immunity suggests that the efficacy of anti-PD-L1 antibody plus paclitaxel synergistic therapy may be improved. We found that anti-PD-L1 antibody alone was modestly effective for TNBC treatment. Combination of paclitaxel with anti-PD-L1 antibody showed an optimal effect on suppressing the growth of tumors formed by Atad3a-knockdown 4T1 cells compared with Atad3a-high cells (Fig. 5n–p; Supplementary information, Fig. S9a). Flow cytometry showed that the proportions of infiltrating CD8^+^, IFNγ^+^CD8^+^ and IFNγ^+^CD4^+^ T cells were enhanced in Atad3a-knockdown tumors with combination therapy (Fig. 5q, r; Supplementary information, Fig. S9b–d), while the proportions of exhausted PD-1^+^TIM-3^+^ cells in both CD8^+^ and CD4^+^ T cells were significantly decreased (Fig. 5s; Supplementary information, Fig. S9e). IHC analysis confirmed that PD-L1 protein level was markedly elevated after combination therapy in Atad3a-abundant tumors rather than in Atad3a-knockdown tumors, likely resulting in prevention of the infiltration of CD8^+^ T cells (Supplementary information, Fig. S9f). In contrast, the number of infiltrating CD8^+^ T cells was significantly increased in Atad3a-knockdown tumors after combination therapy (Supplementary information, Fig. S9f). These results demonstrate that Atad3a-knockdown promotes the efficacy of combination therapy for TNBC.

## Discussion

For TNBC, although chemoimmunotherapy partially improves the efficacy in comparison to monotherapy with antibody against PD-1/PD-L1 axis,^36,37^ a large proportion of patients remain non-responsive to the combination therapy, suggesting a challenge to understand the mechanisms. Here, we demonstrate that loss of mitochondrial distribution of PD-L1 is responsible for the resistance to ICI plus paclitaxel chemoimmunotherapy in patients with TNBC. A decreased mitochondrial localization results in failure of mitophagy-dependent PD-L1 degradation, and thus an excessive accumulation of PD-L1 in total and on cell surface, creating an unfavorable immunosuppressive tumor microenvironment (TME) that reduces the efficacy of immunotherapy.

ATAD3A protein is encoded by nuclear genome and localizes in mitochondria.^38^ In addition to regulation of multiple biological processes including mitochondrial dynamics, homeostasis and metabolism, ATAD3A also plays a vital role in tumor development and progression.^39^ Furthermore, silence of ATAD3A has been reported to increase CD45^+^ TILs as well as memory CD45RO^+^ TILs in colorectal cancer.^40^ In patients with neurological disease, heterozygous mutation in ATAD3A up-regulates interferon-stimulated gene and interferon α protein expression via activating cyclic GMP-AMP synthase and stimulator of interferon genes (cGAS-STING) pathway.^41^ Here, we discovered ATAD3A as a key regulator that prevents PD-L1 mitochondrial distribution. Thus, in preclinical model, targeting ATAD3A to restore mitochondrial localization and degradation of PD-L1 could boost antitumor immunity.

Cancer cells maintain high PD-L1 expression to acquire immune evasion through multiple regulatory mechanisms including transcription regulation, epigenetic regulation, translation regulation, posttranslational modification, cellular distribution regulation and prevention of degradation.^42^ The ubiquitination-mediated or lysosomal pathways of PD-L1 degradation regulated by CMTM6, CMTM4, CDK4 and CSN5 have previously been documented.^43–46^ PD-L1 also undergoes endoplasmic-reticulum-associated degradation through serine195 phosphorylation by AMP-activated protein kinase (AMPK) and autophagic degradation via cargo receptor SQSTM1/p62.^47,48^ Our study reveals a mitophagy-dependent redistribution and subsequent degradation of PD-L1. Mitophagy, as a cargo-specific autophagy, not only selectively removes mitochondria but also associates with protein degradation.^49–51^ We demonstrate that PINK1, a key activator of mitophagy, facilitates the recruitment of PD-L1 to mitochondria as a targeted substrate for degradation. Additionally, we reveal that ATAD3A, a negative regulator of PINK1-dependent mitophagy, prevents PD-L1 distribution at mitochondria and degradation, thus providing new insight into mitochondria-mediated PD-L1 protein homeostasis regulation in tumor immune microenvironment (Supplementary information, Fig. S10). Furthermore, PINK1-dependent mitophagy-mediated distribution and degradation of PD-L1 are associated with beneficial responses to immunotherapy. This is in accordance with the recent report in which optineurin-dependent mitophagy is required for increased therapeutic effect of chemoimmunotherapy by producing CXCL10,^52^ providing additional evidence for the critical involvement of mitophagy in TME immunomodulation.

Chemotherapy drugs show bona-fide immunoregulatory effects during cancer treatment. Some studies demonstrate that certain chemo-drug induced the generation of antitumor immune cells.^53–55^ For example, cisplatin upregulates MHC-I expression on tumor cells and promotes the recruitment and proliferation of effector immune cells.^56^ However, chemotherapy also promotes the formation of immunosuppressive microenvironment favorable for tumor growth. The chemo-drug paclitaxel has been shown to induce proinflammatory responses which promote tumor progression and also blunt the sensitivity to antitumor drugs.^57^ PCB chemotherapy (paclitaxel, carboplatin and bevacizumab combination) increases the proliferation of peripheral blood CD8^+^ effector T cells in non-small cell lung cancer patients. However, the increased T cells express high levels of the immune inhibitory checkpoints PD-1 and CTLA-4 and fail to improve patient survival.^58^ Moreover, a recent study reports that paclitaxel regimen may compromise the efficacy of anti-PD-L1 antibodies in chemoimmunotherapy by reducing antitumor immune cells and increasing immunosuppressive immune cells.^59^ Therefore, understanding the basis for paclitaxel to regulate immunosuppressive tumor microenvironment is critical for developing strategies to overcome the resistance to paclitaxel plus ICI treatment. Our study shows that paclitaxel chemotherapy reduces mitochondrial localization and mitophagy-mediated degradation of PD-L1, resulting in its excessive accumulation in TNBC cells, thus providing a mechanistic basis for paclitaxel to induce immunosuppression and therapy resistance. Furthermore, according to our data, microtubule inhibitors such as nocodazole and colchicine might also upregulate ATAD3A level to control PD-L1 localization.

Our findings in this study suggest that the maintenance of proteostasis and proper level of PD-L1 in tumor cells is critical for the efficacy of ICI treatment, since excessive PD-L1 molecules exhaust an antitumor immune microenvironment and abrogate the effect of blocking antibodies to evoke positive immunity. In support of our observations, a phase 3 KEYNOTE-355 trial showed that in pembrolizumab-chemotherapy, a median progression-free survival for patients with PD-L1 combined positive score (CPS) of 1 or more was 7.6 months compared to 9.7 months for patients with CPS of 10 or more. However, for patients with CPS of 20 or more, the survival was 9.5 months, showing no further benefit.^60^ A phase 1b GO30140 trial showed that the median progression-free survival in patients with PD-L1 expressing score of 10% or more on tumor-infiltrating immune cells or on tumor cells was shorter than that of patients with PD-L1 expressing score less than 10% after atezolizumab treatment (2.7 months vs 3.4 months).^61^ In addition, small-molecule agents targeting PD-L1 as well as its regulatory mechanisms including inhibitors for EGFR (gefitinib), mTOR (rapamycin), ATR (BAY1895433) and NIMA-related kinase 2 (NEK2) downregulate PD-L1 to sensitize tumor cells to combined therapy with anti-PD-1/PD-L1 antibodies.^62–65^ Therefore, excessive PD-L1 may represent an unfavorable TME that is difficult to reverse for sustained therapeutic benefit.

The identification of sensitive versus resistant subpopulations of cancer patients is important for determining the selection of immunotherapy strategies. Studies evaluating biomarkers including PD-L1, tumor mutation burden, counts of TILs and tumor-infiltrating CD8^+^ T cells for predicting responses of patient to therapy demonstrated disappointing limitations.^9,27,66–71^ Our study proposes a concept that PD-L1-positive patients may respond differently to ICI plus paclitaxel combined therapy based on the subcellular redistribution of PD-L1 particularly the mitochondrial localization in tumor cells. We discovered that ATAD3A functions as a key regulator of mitochondrial distribution and degradation of PD-L1. Although our in vitro data showed that the level of ATAD3A could be upregulated by paclitaxel in TNBC cell lines, a small portion of patients with TNBC still maintained the low level of ATAD3A pre- or post-paclitaxel treatment. ATAD3A-low tumor in vivo might overwhelm paclitaxel effect via some suppressive mechanisms, which would be investigated in the future. For instance, some studies have revealed that ATAD3A could be regulated by mTOR, miR-210-5p or be folded properly by S100B.^39,72,73^ Our study indicated that patients with ATAD3A-low TNBC are sensitive to ICI combined with paclitaxel, whereas for those with high-ATAD3A level, the inhibition of ATAD3A is required to increase the beneficial clinical outcome of anti-PD-1/PD-L1 antibodies plus paclitaxel. Although ATAD3A inhibitor is not available currently, some studies have reported some chemotherapy drugs for their negative roles in regulating PD-L1 expression, which might be more practicable to combine with anti-PD-1/PD-L1 antibodies for ATAD3A-high TNBC patients with CM-signature.^74–76^ Thus, we have demonstrated a potential strategy of personalized management of TNBC that overcomes immunosuppression and resistance to ICI in combination with paclitaxel (Fig. 5t).

## Materials and methods

### Cell culture

Human triple-negative breast cancer cell lines MDA-MB-231, BT549, MDA-MB-468, mouse breast cancer cell line 4T1, and human embryonic kidney HEK293T cells were purchased from the American Type Culture Collection and were grown in a humidified incubator at 37 °C in 5% CO_2_. MDA-MB-231, MDA-MB-468, and HEK293T cells were maintained in DMEM supplemented with 10% FBS (Gibco). BT549 and 4T1 cells were cultured in RPMI-1640 medium with 10% FBS. Both media were supplemented with 100 units/mL penicillin, 100 µg/mL streptomycin (Gibco). All cell lines were routinely verified to be mycoplasma negative using a mycoplasma detection kit (Lonza).

### Plasmids

shRNA lentiviral vectors used were as follows:

shATAD3A#1(human): 5’-TAGCAACAAGGAACACCAA-3’;

shATAD3A#2(human): 5’-CCTGAGTCCACAGGGAGAT-3’;

shPINK1#1(human): 5’-CCTAACCGTCTCCGCTTCTTC-3’;

shPINK1#2(human): 5’-CGGCTGGAGGAGTATCTGATA-3’;

shAtad3a#1(mouse): 5’-TGTTGGAGTCTATTCTGCAAAGAAT-3’;

shAtad3a#2(mouse): 5’-GCCTGTATAGGAACGTTCTGATGTA-3’;

shPink1#1(mouse): 5’-GGCTGGAGAGTATGGAGCAGTTACT-3’;

shPink1#2(mouse): 5’-CATCCTTGTGGAGTGGGACTCAGAT-3’;

For in vitro protein interaction analysis, full length PINK1 (amino acids 1–581) and truncated PINK1 (amino acids 1–155, amino acids 156–320, amino acids 321–509, amino acids 510–581) were generated and subcloned into pZDonor-3Flag vector.

### Human tissue samples

The chemotherapy trial was from the southwest hospital containing 15 TNBC patients with TE regimen (paclitaxel/docetaxel, epirubicin) or TEC regimen (docetaxel, epirubicin and cyclophosphamide) for neoadjuvant chemotherapy. The biopsies were taken at their first to doctor as pre-chemotherapy samples and the intraoperative specimens were taken after the neoadjuvant treatment as post-chemotherapy samples. The immunotherapy cohort was from the FUTURE trial C arm (NCT03805399). These patients were with locally advanced or metastatic TNBC progressed after standard chemotherapy including taxol. Intraoperative specimens were taken in the radical mastectomy or modified mastectomy as pre-chemotherapy samples. With standard chemotherapy failure, the progressive and unresectable tumor biopsies were taken as post-chemotherapy samples before being treated with nab-paclitaxel plus anti-PD-1 antibodies, which were regarded as the baseline of chemoimmunotherapy. For the survival analysis, we collected 399 breast cancer patients for the multicentric clinical cohort from Chongqing (147 cases), Guangzhou (89 cases) and Beijing (163 cases) trials. Most patients have undergone radical mastectomy or modified mastectomy and have received the standard chemotherapy. All procedures were approved by the Ethics Committee of the First Affiliated Hospital of Army Medical University (KY20200305). Informed consent was obtained from all patients.

### IHC staining and evaluation

IHC staining was performed on 4 μm formalin-fixed paraffin-embedded tissue sections, which were incubated at 60 °C for 1 h and deparaffinized and rehydrated with xylene and graded alcohol. For antigen retrieval, the sections were performed with EDTA (pH 8.0) or citrate buffer (pH 6.0) at high temperature and pressure for 3 min and cooled down to room temperature. Tissue sections were blocked with 3% hydrogen peroxide methanol solution for 30 min at 37 °C to inactivate the endogenous peroxidase and then added with animal nonimmune serum for 30 min in a humid chamber at 37 °C, followed by incubation with the primary antibodies overnight at 4 °C, including anti-ATAD3A (NBP2-14881, Novus Biologicals), anti-PD-L1 (clone 28-8, Abcam; D5V3B, Cell Signaling Technology), anti-CD8a (D4W2Z, Cell Signaling Technology), anti-CD8 (clone C8/144B, MXB Biotechnologies), anti-granzyme B (D6E9W, Cell Signaling Technology), anti-Ki67 (clone SP6, Abcam). After washing with PBS three times, the sections were incubated with secondary antibody (Dako) for 30 min in a humid chamber at 37 °C. The sections were washed with PBS for three times and performed with Dako Real™ Envison kit for staining. IHC score was quantified using Image-Pro Plus software 6.0, determined by integrated optical density (IOD) value/area, three to five fields per section.

### Flow cytometry

For cell surface PD-L1 analysis, human or mouse breast cancer cells were stained with anti-human CD274 (APC, clone 29E.2A3, BioLegend) or anti-mouse CD274 (APC, clone 10 F.9G2, BioLegend) at 1:100 dilution for 30 min at 4 °C and washed with PBS plus 2% FBS and 2 mM EDTA (FACS buffer). Mouse IgG2b (APC, clone MPC-11, BioLegend) and rat IgG2b (APC, clone RTK4530, BioLegend) were used as controls respectively. Stained cells were analyzed by LSRFortessa cytometer plus BD DIVA or FlowJo software.

For the analysis of immunophenotype of orthotopic tumors, tumor tissues were digested in DF12 containing collagenase I, collagenase IV, DNase I (Sigma-Aldrich) for 1 h at 37 °C. Then digested tissues were filtered through a 70 μm mesh strainer, followed by incubation in red blood cell lysis buffer for 3 min at 4 °C. After washing twice with FACS buffer, 1 × 10^6^ cells were stained with Fixable Viability Dye eFluor™ 780 (eBioscience). After washing with FACS buffer, cells were stained with appropriate surface marker antibodies at 4 °C for 30 min, including anti-CD3 (PE, clone 145-2C11, BioLegend), anti-CD45.2 (Brilliant Violet 510, clone 104, BioLegend), anti-CD4 (PE/Cyanine7, clone RM4-5, BioLegend; FITC, clone GK1.5, BD Biosciences), anti-CD8 (Brilliant Violet 605, clone 53–6.7, BioLegend), anti-CD25 (PerCP/Cyanine5.5, clone PC61, BioLegend), anti-PD-1(PE/Cyanine7, clone RMP1-30, BioLegend), anti-TIM-3 (Brilliant Violet 421, clone RMT3-23, BioLegend), anti-Ly-6G (FITC, clone 1A8, BD Biosciences), anti-CD11b (PE, clone M1/70, BD Biosciences), anti-F4/80 (APC, clone BM8, BioLegend), anti-NK-1.1 (PerCP/Cyanine5.5, clone PK136, BioLegend). Intracellular staining for Foxp3 (APC, clone FJK-16s, eBioscience) or IFNγ (FITC, clone XMG1.2, BioLegend) was performed after fixation and permeabilization (Transcription Factor Staining Buffer Set, eBioscience; Fixation/Permeabilization Solution Kit, BD Biosciences). Stained cells were analyzed by LSRFortessa cytometer plus BD DIVA or FlowJo software.

### Analysis of mitochondrial mass and dysfunctional mitochondria

TNBC cells were treated with paclitaxel for 16 h. Then cells were incubated with 50 nM MitoTracker Green FM (Invitrogen) and 100 nM MitoTracker Red CMXRos (Invitrogen) at 37 °C for 20 min and were analyzed by flow cytometry.

### Immunofluorescence

For cell immunofluorescence, 2 × 10^4^ cells were seeded on each confocal dish and cultured for 1 day. Cells were fixed with 4% paraformaldehyde for 15 min at room temperature. After permeabilizing with 0.1% Triton X-100 or cold digitonin (Abcam) for 10 min and blocking with normal goat serum for 30 min, samples were incubated with primary antibodies overnight at 4 °C, including anti-TOM20 (clone F-10, Santa Cruz Biotechnology; BM4366, Boster), anti-PD-L1 (clone EPR19759, Abcam; LS-C754760, LifeSpan BioSciences; 2B11D11, Proteintech), anti-LAMP1 (clone D2D11, Cell Signaling Technology; clone 1D4B, Santa Cruz Biotechnology), anti-TGN46 (clone 1F6D5, Proteintech), anti-Calnexin (clone 2A2C6, Proteintech). The cells were washed with PBS-T for three times and incubated with secondary antibodies (Alexa Fluor Plus 555, Alexa Fluor Plus 488, Alexa Fluor Plus 647, Invitrogen) at room temperature for 1 h. Antifade Mounting Medium with DAPI (Beyotime) was used to mount the samples. The confocal images were then taken on Zeiss LSM 900 and Airyscan 2 confocal laser scanning microscope and analyzed with ZEN 2.3 software.

For MitoTracker staining, cells were incubated with MitoTracker Deep Red dyes in complete medium at 37 °C for 30 min, then were removed of excess unbound dyes by washing twice.

For tissue immunofluorescence, paraffin sections of patients with TNBC were baked at 60 °C for 30 min followed by deparaffinization and rehydration using xylene and graded ethanol. After EDTA (pH 8.0) antigen retrieval and endogenous peroxidase blockade as IHC staining methods mentioned above, the samples were permeabilized with 0.3% Triton for 30 min and blocked with normal goat serum for 30 min at room temperature. Primary antibodies targeting PD-L1, LAMP1 and TOM20 were incubated with samples overnight at 4 °C. After secondary antibody incubation for 1 h and DAPI incubation for 15 min, 0.3% Sudan black-B solution was used to quench spontaneous fluorescence for 20 min at room temperature followed by washing twice. The confocal images were then taken and analyzed.

### Quantitative reverse-transcription PCR assays

Total RNAs were isolated with RNA Isolation Kit. 2 μg of total RNA was converted into cDNA using the PrimeScript RT Master Perfect Real Time Kit (TaKaRa, Japan) according to the manufacturer’s instructions and subjected to RT-PCR. Primers used were as follows:

Human ATAD3A, forward, 5’-CGCCATAGCAACAAGGAACACC-3’;

reverse, 5’-ATGGCGTAGTCCATGCCTGAGT-3’;

Mouse Atad3a, forward, 5’-GGAGCTGAGGCATAAAAACGA-3’;

reverse, 5’-TCGAATCTGTTCCCGGATGAT-3’;

Human PD-L1, forward, 5’-GCTGCACTAATTGTCTATTGGGA-3’;

reverse, 5’-AATTCGCTTGTAGTCGGCACC-3’;

Mouse PD-L1, forward, 5’-GACGCAGGCGTTTACTGCT-3’;

reverse, 5’-GCGGTATGGGGCATTGACTTT-3’;

Human ACTB, forward, 5’-GCAAAGACCTGTACGCCAACA-3’;

reverse, 5’-TGCATCCTGTCGGCAATG-3’;

Mouse ACTB, forward, 5’-GGCTGTATTCCCCTCCATCG-3’;

reverse, 5’-CCAGTTGGTAACAATGCCATGT-3.

### Immunoblot and immunoprecipitation

For immunoblot analysis, cells were collected when cell culture reached 70% confluence. Total cells were washed with PBS for three times and lysed in RIPA buffer (Thermo Fisher Scientific) supplemented with protease inhibitor cocktail (Roche) and phosphatase inhibitor tablets (Roche) on ice for 30 min. For mitochondria isolation analysis, mitochondria were isolated with a Mitochondria Isolation Kit (Thermo Fisher Scientific) and lysed in mitochondrial lysis buffer (Beyotime). Cell lysates or mitochondria lysates were centrifuged at 13,000× *g* for 15 min to harvest protein supernatant and concentrations were determined by BCA assay (Thermo Fisher Scientific). Equal amounts of proteins were subjected to 10% SDS-PAGE and transferred to PVDF membranes (Bio-Rad). PVDF membranes were blocked in 5% milk dissolved in PBS + 0.1% Tween-20 for 1 h and were incubated with the indicated primary antibodies overnight at 4 °C, including anti-PD-L1 (clone E1L3N, Cell Signaling Technology; clone 405.9A11, Cell Signaling Technology, clone 2096 C, R&D), anti-β-Actin (clone 8H10D10, Cell Signaling Technology), anti-PINK1 (BC100-494, Novus Biologicals), anti-ATAD3A (NBP2-14881, Novus Biologicals; LS-B13967, LifeSpan BioSciences), anti-TOM20 (clone D8T4N, Cell Signaling Technology), anti-DYKDDDDK (clone D6W5B, Cell Signaling Technology), anti-Tubulin (clone D3U1W, Cell Signaling Technology), anti-Biotin (clone D5A7, Cell Signaling Technology). With washing three times, the PVDF membranes were incubated with secondary antibodies (Rockland; Beyotime) conjugated with horseradish peroxidase (HRP) for 1 h followed by electrochemiluminescence (ECL) to analyze the protein expression.

For immunoprecipitation, HEK293T cells were lysed in IP Lysis buffer (Thermo Fisher Scientific) supplemented with protease inhibitor cocktail (Roche) and phosphatase inhibitor tablets (Roche) on ice for 15 min. After centrifuging, cell lysates were incubated with antibodies against PINK1 (clone 38CT20.8.5, Santa Cruz Biotechnology) or PD-L1 (LS‑C754760, LifeSpan BioSciences) overnight at 4 °C, followed by incubation with 40 μL Protein G Sepharose beads (GE Healthcare) for 3 h at 4 °C. Then the beads were washed six times with IP lysis buffer and subjected to immunoblot analysis.

### EM imaging for PD-L1-APEX2

BT549 cells infected with PD-L1-APEX2 fusion lentiviral were grown on glass coverslips placed inside six-well plates. Then cells were fixed with 2% glutaraldehyde for 2 h and performed with DAB staining. Post-fixation staining was carried out with chilled 1% osmium tetroxide (Electron Microscopy Sciences) for 8 min. Cells were rinsed with chilled distilled water. After washing in distilled water, cells were dehydrated in a graded ethanol series (70%, 95%, 100%, 100%) and acetone for 10 min each time and infiltrated in EMBED-812 (Electron Microscopy Sciences). Embedded cells were carefully cut with a diamond knife into 70 nm ultrathin sections. Images were captured on a 120 kV electron microscope (Tecnai Spirit, FEI) at 100 kV with an CCD camera (MoradaG3, EMSIS) using RADIUS (EMSIS) software.

### In vitro binding assay

Flag-labeled full-length and truncated PINK1 were expressed in HEK293T cells and purified using 3× Flag peptide (Sigma), followed by incubation with Biotin-labeled PD-L1 ICD peptide (amino acids 262–290, GenScript) in the buffer containing 25 mM Tris-HCl pH 7.4, 150 mM NaCl, 1 mM EDTA, 0.05% NP-40 and 10% glycerol supplemented with protease inhibitors (Roche) and phosphatase inhibitor tablets (Roche) for 12 h at 4 °C. Then we added streptavidin sepharose beads (Cell Signaling Technology) or anti-Flag M2 affinity gel (Sigma) to in vitro mixture for incubation for 1.5 h. After washing with IP lysis buffer (Thermo Fisher Scientific) for five times, the pull-down complex was subjected to immunoblot analysis.

### Mouse experiments

Animal experiments were approved by the Institutional Animal Care and Use Committee at Army Medical University (AMUWEC20201344). For the immunodeficiency mouse model, 4T1 tumors formed by control or Atad3a-knockdown cells (1 × 10^5^ cells in 50 μL PBS mixed with 50 μL Matrigel, Corning) were injected into the fourth left mammary fat pads of female BALB/c nude mice. For the immune-competent mouse model, 4T1 tumors formed by control or Atad3a-knockdown cells (5 × 10^4^ cells in 50 μL PBS mixed with 50 μL Matrigel, Corning) were injected into the fourth left mammary fat pads of female BALB/c mice. For in vivo treatment, mice were treated randomly with paclitaxel (8 mg/kg, HY-B0015, MedChemExpress) or vehicle intraperitoneally on 5, 8, 11, and 14 days after tumor cells inoculation, while mouse anti-PD-L1 antibody (100 μg per mouse, clone 10 F.9G2, BE0101, BioXcell) or rat IgG2b isotype control (100 μg per mouse, clone LTF-2, BE0090, BioXcell) was utilized on 6, 9, 12, 15 days after inoculation. Orthotropic tumor sizes were measured every 4 days with a caliper and tumor volumes = Length × Width^2^/2 were calculated.

### Statistical analysis

Statistical analysis was performed using SPSS 23.0 and GraphPad Prism 6.0. Data were presented as means ± SD and *P*-value less than 0.05 was considered statistically significant. * indicates *P* < 0.05, ** indicates *P* < 0.01, *** indicates *P* < 0.001, ns indicates no significance. A two-sided *t*-test was used to compare two independent groups. One-way ANOVA was used for more than two-group comparisons. The Mann-Whitney U test was used to compare the difference in expression level of ATAD3A. Kaplan-Meier analysis was used to assess survival curves of different groups and *P*-value was calculated by log-rank test. The Median was used to determine the cut-off point. Wilcoxon rank-sum test or Fisher’s exact test was used to analyze the correlation between ATAD3A expression and clinicopathological parameters.

## Supplementary information

Fig. S1

Fig. S2

Fig. S3

Fig. S4

Fig. S5

Fig. S6

Fig. S7

Fig. S8

Fig. S9

Fig. S10

Supplementary video legend

Video-S1

Video-S2

Video-S3

Video-S4
