# Supplementary material for: Targeting ATAD3A-PINK1-mitophagy axis overcomes chemoimmunotherapy resistance by redirecting PD-L1 to mitochondria
Source: Cell Res. 2023 Jan 10;33(3):215–28. doi: 10.1038/s41422-022-00766-z (PMC9977947; doi:10.1038/s41422-022-00766-z)
Supplement: Supplementary file 1 — Fig. S1 [file 41422_2022_766_MOESM1_ESM.pdf]

Fig. S1

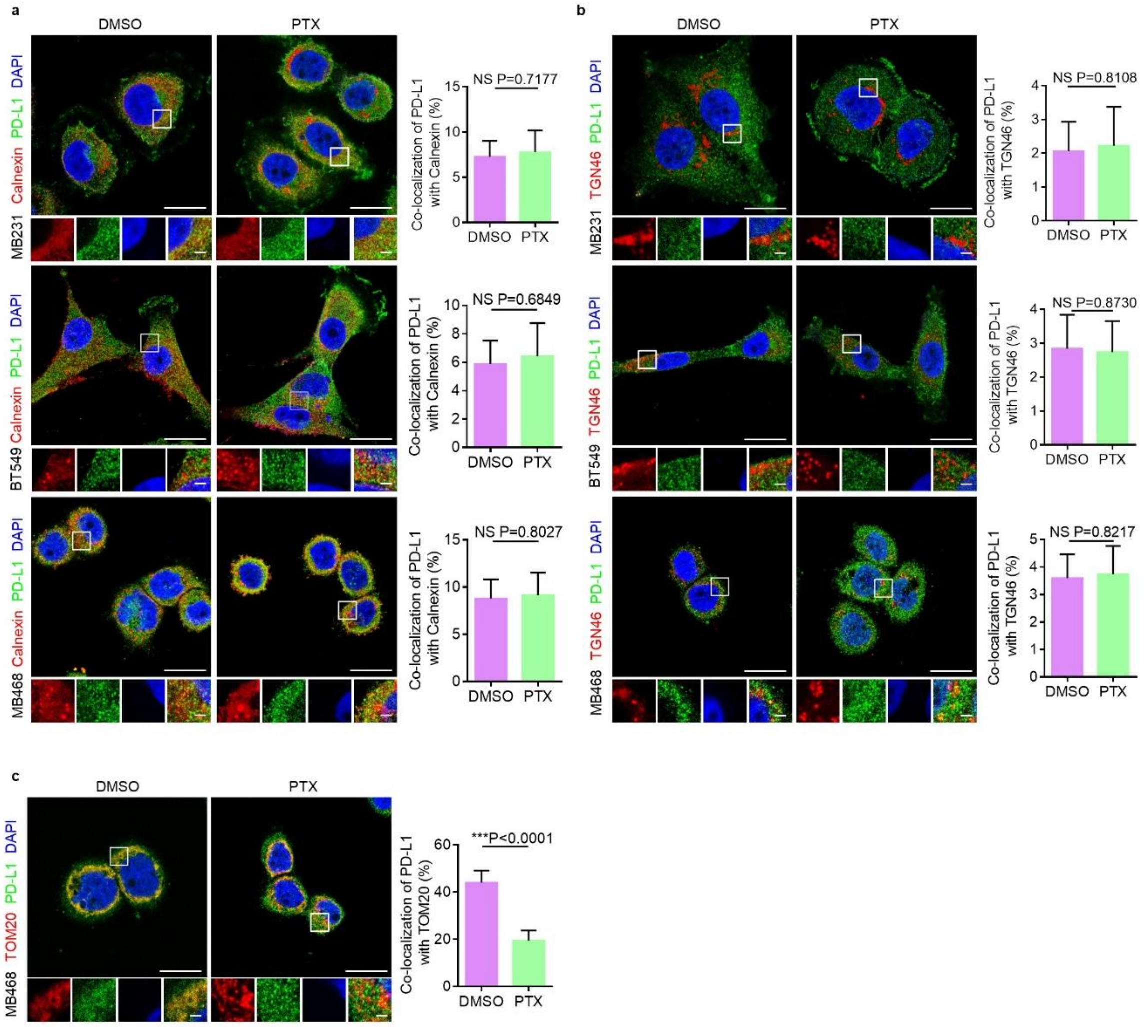

Fig. S1 The effect of paclitaxel on subcellular localization of PD-L1.

a Left, immunostaining of PD-L1 (green) and Calnexin-labeled endoplasmic reticulum (red) in TNBC cells with or without 20 nM paclitaxel treatment for 12 h in TNBC cells. Scale bars, 20 μm and 2 μm (inset). Right, the percentage of PD-L1 co-localized with Calnexin (*n* = 5 fields, *t*-test). **b** Left, immunostaining of PD-L1 (green) and TGN46-labeled Golgi complex (red) with or without 20 nM paclitaxel treatment for 12 h in TNBC cells. Scale bars, 20 μm and 2 μm (inset). Right, the percentage of PD-L1 co-localized with TGN46 (*n* = 5 fields, *t*-test). **c** Left, immunostaining of PD-L1 (green) and TOM20-labeled mitochondria (red) with or without 20 nM paclitaxel treatment for 12 h in MDA-MB-468 cells. Scale bars, 20

μm and 2 μm (inset). Right, the percentage of PD-L1 co-localized with TOM20 (n = 5 fields, t-test). Data are representative of at least two independent experiments and are shown as means ± SD.
