# Supplementary material for: Targeting ATAD3A-PINK1-mitophagy axis overcomes chemoimmunotherapy resistance by redirecting PD-L1 to mitochondria
Source: Cell Res. 2023 Jan 10;33(3):215–28. doi: 10.1038/s41422-022-00766-z (PMC9977947; doi:10.1038/s41422-022-00766-z)
Supplement: Supplementary file 2 — Fig. S2 [file 41422_2022_766_MOESM2_ESM.pdf]

Fig. S2

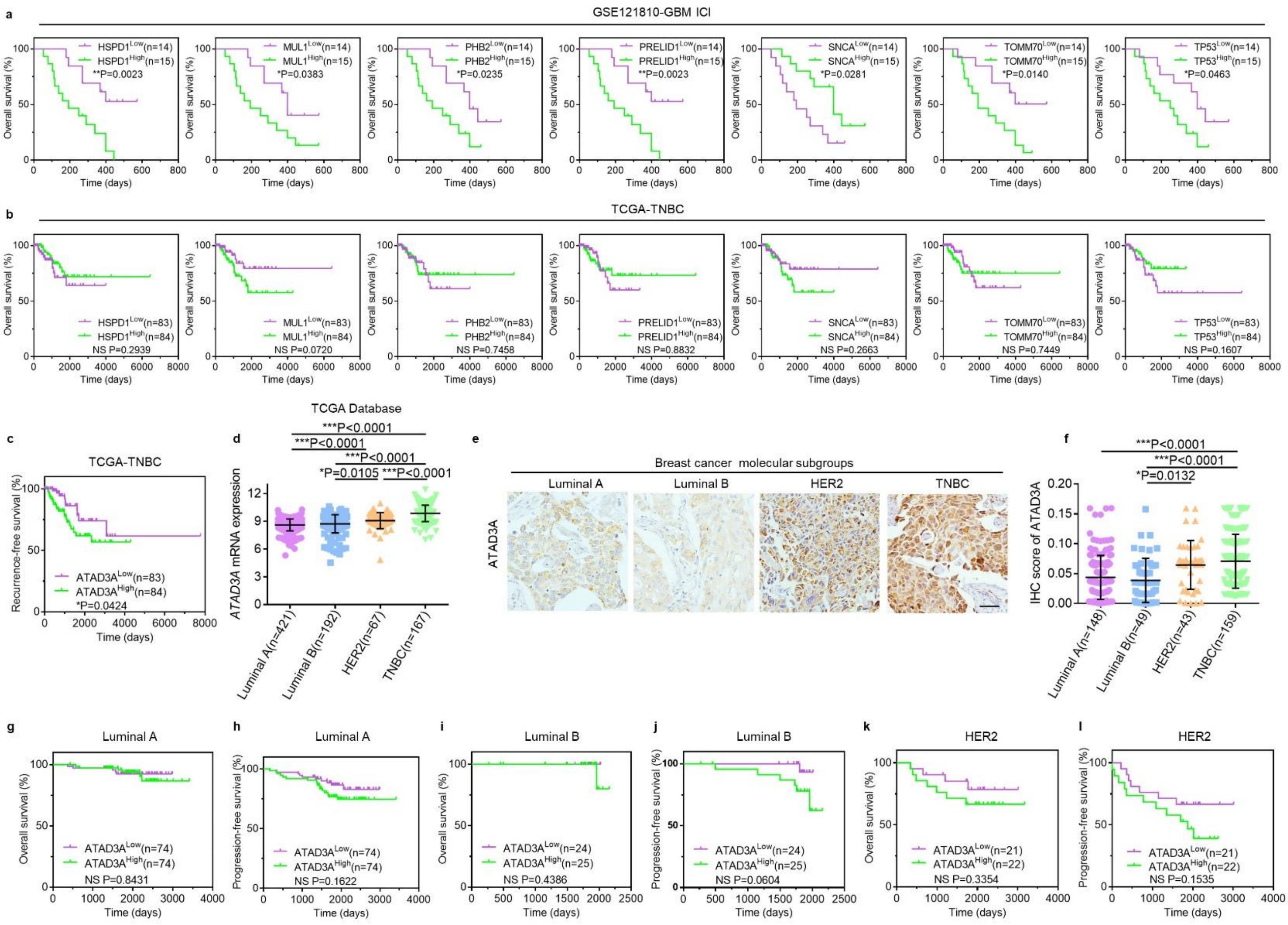

Fig. S2 The expression of ATAD3A and other candidate genes in the immunotherapy cohort and breast cancer cohort.

a Kaplan-Meier analysis of the overall survival of glioblastoma patients who received anti-PD-1 antibody treatment with high or low mRNA expression of candidate genes in tumors (n = 29). Data were derived from GSE121810 cohort. Cutoff of candidate gene expression was determined by the median (Kaplan-Meier method and log-rank test). **b** Kaplan-Meier analysis of the overall survival of TNBC patients with high or low level of candidate genes. Data were derived from TCGA database. Cutoff was

determined by the median (Kaplan-Meier method and log-rank test). **c** Kaplan-Meier analysis of the recurrence-free survival of TNBC patients with high or low ATAD3A expression. Data were derived from TCGA database. Cutoff was determined by the median (Kaplan-Meier method and log-rank test). **d** Quantification of ATAD3A mRNA in luminal A (n = 421), luminal B (n = 192), HER2-overexpressing (n = 67) and TNBC (n = 167). Data were derived from TCGA database (one-way ANOVA). **e, f** Representative images (**e**) and quantification (**f**) of ATAD3A staining in tumor area on serial sections from luminal A (n = 148), luminal B (n = 49), HER2 (n = 43) and TNBC (n = 159). The data were derived from 399 breast cancer patients in the multicentric clinical cohort of this study (one-way ANOVA). These patients underwent radical mastectomy or modified mastectomy and received the standard chemotherapy. Scale bars, 50 μm. **g**, **h** Kaplan-Meier analysis of the overall survival (**g**) and progression-free survival (**h**) of luminal A breast cancer patients with high or low ATAD3A expression. Cutoff of ATAD3A expression was determined by the median (Kaplan-Meier method and log-rank test). **i**, **j** Kaplan-Meier analysis of the overall survival (**i**) and progression-free survival (**j**) of luminal B breast cancer patients with high or low ATAD3A expression (Kaplan-Meier method and log-rank test). **k**, **l** Kaplan-Meier analysis of the overall survival (**k**) and progression-free survival (**l**) of patients with HER2-overexpressing breast cancer with high or low ATAD3A expression (Kaplan-Meier method and log-rank test).
