# Supplementary material for: Targeting ATAD3A-PINK1-mitophagy axis overcomes chemoimmunotherapy resistance by redirecting PD-L1 to mitochondria
Source: Cell Res. 2023 Jan 10;33(3):215–28. doi: 10.1038/s41422-022-00766-z (PMC9977947; doi:10.1038/s41422-022-00766-z)
Supplement: Supplementary file 3 — Fig. S3 [file 41422_2022_766_MOESM3_ESM.pdf]

Fig. S3

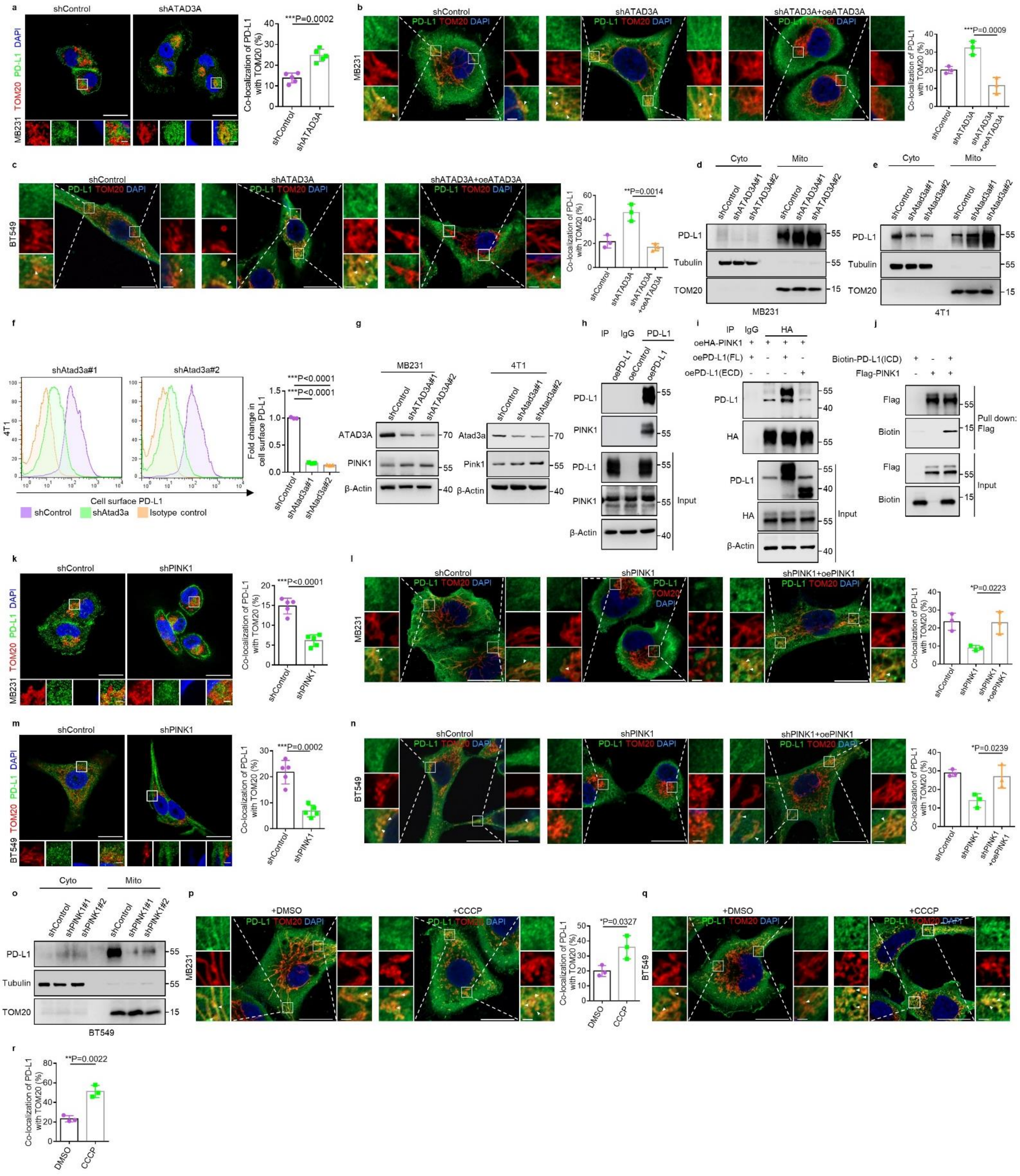

Fig. S3 ATAD3A-PINK1 axis regulates the mitochondrial localization of PD-L1.

a Left, immunostaining of PD-L1 (green) and TOM20-labeled mitochondria (red) in MDA-MB-231 cells with or without ATAD3A-knockdown. Scale bars, 20 μm and 2 μm (inset). Right, the percentage of PD-L1 co-localized with TOM20 (*n* = 5 fields, *t*-test). **b**, **c** Left, immunostaining of PD-L1 (green) and TOM20-labeled mitochondria (red) in MDA-MB-231 cells (**b**) and BT549 cells (**c**) with ATAD3A-knockdown or ATAD3A-knockdown plus ATAD3A-overexpression. Scale bars, 20 μm and 2 μm (inset). Right, the percentage of PD-L1 co-localized with TOM20 (*n* = 3 fields, one-way ANOVA). **d**, **e** Immunoblot of PD-L1 in the cytoplasm and mitochondria of control and ATAD3A-knockdown MDA-MB-231 cells (**d**) and 4T1 cells (**e**). TOM20 and Tubulin were used as mitochondria and cytoplasm protein controls. Cyto, cytoplasm, mito, mitochondria. **f** Flow cytometry (left) and quantification (right) of surface PD-L1 in control and Atad3a-knockdown 4T1 cells with 500 IU/mL IFNγ treatment (*n* = 3, one-way ANOVA). **g** Immunoblot of PINK1 in control and ATAD3A-knockdown MDA-MB-231 cells (left) and 4T1 cells (right). **h** Immunoblot of PD-L1 and PINK1 in HEK293T cells overexpressing PD-L1 (OE-PD-L1) and control cells (OE-Control), assessed after immunoprecipitation with immunoglobulin G (IgG) or antibody to PD-L1. **i** Immunoblot of PD-L1 and HA-tag in HEK293T overexpressing HA-tag-PINK1, full length of PD-L1 (FL) and extracellular domain of PD-L1 (ECD), assessed after immunoprecipitation with immunoglobulin G (IgG) or antibody to HA-tag. **j** Protein direct interaction analysis of the intracellular domain of PD-L1 (ICD) and PINK1 in vitro. Purified Flag-labeled full-length PINK1 was incubated with Biotin-labeled PD-L1 ICD domain, followed by Flag pull-down and

immunoblot. **k**, **m** Left, immunostaining of PD-L1 (green) and TOM20-labeled mitochondria (red) in control and PINK1-knockdown MDA-MB-231 cells (**k**) or BT549 cells (**m**). Scale bars, 20 μm and 2 μm (inset). Right, the percentage of PD-L1 co-localized with TOM20 (n = 5 fields, t-test). **l**, **n** Left, immunostaining of PD-L1 (green) and TOM20-labeled mitochondria (red) in MDA-MB-231 cells (**l**) and BT549 cells (**n**) with PINK1-knockdown or PINK1-knockdown plus PINK1-overexpression. Scale bars, 20 μm and 2 μm (inset). Right, the percentage of PD-L1 co-localized with TOM20 (n = 3 fields, one-way ANOVA). **o** Immunoblot of PD-L1 in the cytoplasm and mitochondria of BT549 cells with or without PINK1-knockdown. TOM20 and Tubulin were used as mitochondria and cytoplasm protein controls. Cyto, cytoplasm, mito, mitochondria. **p** Left, co-localization of PD-L1 (green) and TOM20-labeled mitochondria (red) with or without 20 μM CCCP treatment for 3 h in MDA-MB-231 cells. Arrowheads, co-localization. Scale bars, 20 μm and 2 μm (inset). Right, the percentage of PD-L1 co-localized with TOM20 (n = 3 fields, t-test). **q** Co-localization of PD-L1 (green) and TOM20-labeled mitochondria (red) with or without 20 μM CCCP treatment for 3 h in BT549 cells. Arrowheads, co-localization. Scale bars, 20 μm and 2 μm (inset). **r** The percentage of PD-L1 co-localized with TOM20 in BT549 cells (n = 3 fields, t-test). Data are representative of at least three independent experiments and are shown as means ± SD.
