# Supplementary material for: Targeting ATAD3A-PINK1-mitophagy axis overcomes chemoimmunotherapy resistance by redirecting PD-L1 to mitochondria
Source: Cell Res. 2023 Jan 10;33(3):215–28. doi: 10.1038/s41422-022-00766-z (PMC9977947; doi:10.1038/s41422-022-00766-z)
Supplement: Supplementary file 4 — Fig. S4 [file 41422_2022_766_MOESM4_ESM.pdf]

Fig. S4

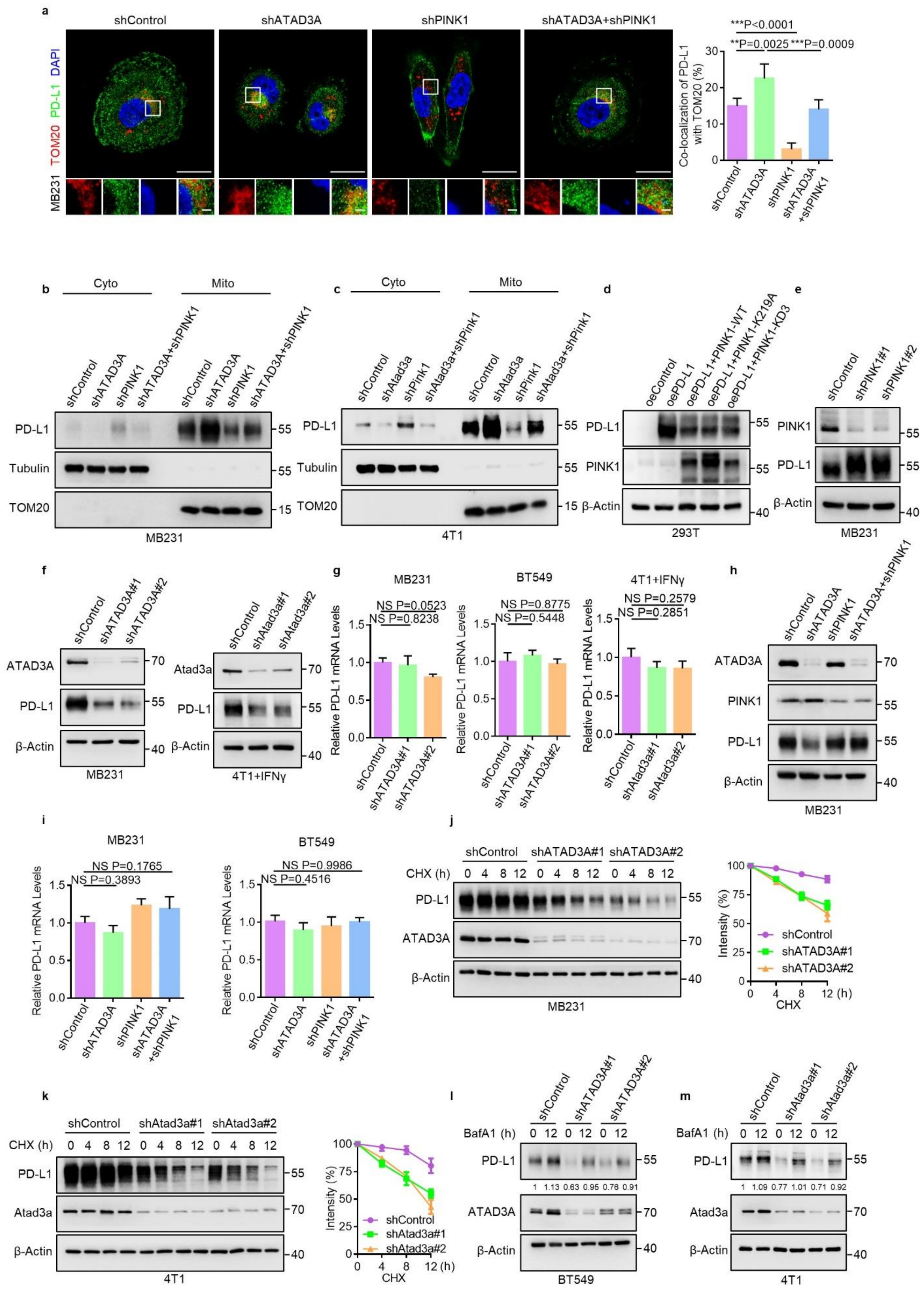
a
shControl
shATAD3A
shPINK1
shATAD3A+shPINK1
MB231 TOM20 PD-L1 DAPI
Co-localization of PD-L1 with TOM20 (%)
***P<0.0001
**P=0.0025
***P=0.0009
b
Cyto
Mito
PD-L1
Tubulin
TOM20
MB231
c
4T1
d
oeControl
oePD-L1
oePD-L1+PINK1-WT
oePD-L1+PINK1-K219A
oePD-L1+PINK1-KD3
PINK1
β-Actin
293T
e
shPINK1#1
shPINK1#2
f
shATAD3A#1
shATAD3A#2
ATAD3A
Atad3a
4T1+IFNγ
g
MB231
BT549
4T1+IFNγ
Relative PD-L1 mRNA Levels
NS P=0.0523
NS P=0.8238
NS P=0.8775
NS P=0.5448
NS P=0.2579
NS P=0.2851
h
i
NS P=0.1765
NS P=0.3893
NS P=0.9986
NS P=0.4516
j
CHX (h)
Intensity (%)
k
shAtad3a#1
shAtad3a#2
l
BafA1 (h)
1 1.13 0.63 0.95 0.76 0.91
m
1 1.09 0.77 1.01 0.71 0.92

Fig. S4 ATAD3A-PINK1 mitophagy axis plays a crucial role in regulating PD-L1 degradation.

a Left, immunostaining of PD-L1 (green) and TOM20 (red) in control, ATAD3A-knockdown, PINK1-knockdown or ATAD3A and PINK1 double knockdown MDA-MB-231 cells. Scale bars, 20 μm and 2 μm (inset). Right, the percentage of PD-L1 co-localized with TOM20 (n = 5 fields, one-way ANOVA). **b**, **c** Immunoblot of PD-L1 in the cytoplasm and mitochondria of control, ATAD3A-knockdown, PINK1-knockdown or ATAD3A and PINK1 double knockdown MDA-MB-231 cells (**b**) and 4T1 cells (**c**). TOM20 and Tubulin were used as mitochondria and cytoplasm protein controls. Cyto, cytoplasm, mito, mitochondria. **d** Immunoblot of indicated proteins in PD-L1 transfected HEK293T cells with overexpression of wild-type PINK1 or kinase-dead PINK1 (K219A mutant or K219A, D362A and D384A triple kinase-dead mutant). **e** Immunoblot of PD-L1 in control and PINK1-knockdown (shPINK1#1 and shPINK1#2) MDA-MB-231 cells. **f** Immunoblot of PD-L1 in MDA-MB-231 cells (left) and 4T1 cells (right) with or without ATAD3A-knockdown. 4T1 cells were treated with 500 IU/mL IFNγ for 24 h. **g** qRT-PCR of PD-L1 mRNA in control, ATAD3A-knockdown MDA-MB-231 cells, BT549 cells and 4T1 cells. 4T1 cells were treated with 500 IU/mL IFNγ for 24 h (n = 3, one-way ANOVA). **h** Immunoblot of total PD-L1 in control, ATAD3A-knockdown, PINK1-knockdown or ATAD3A and PINK1 double knockdown MDA-MB-231 cells. **i** qRT-PCR of PD-L1 mRNA in control, ATAD3A-knockdown, PINK1-knockdown or ATAD3A and PINK1 double knockdown MDA-MB-231 cells and BT549 cells. **j**, **k** Left, immunoblot of PD-L1 in

control and ATAD3A-knockdown MDA-MB-231 cells (**j**) and 4T1 cells (**k**) treated with 20 μM cycloheximide (CHX) for indicated times. h, hours. Right, quantification of PD-L1 intensity detected in immunoblot. **l**, **m** Immunoblot of PD-L1 in control and ATAD3A-knockdown BT549 cells (**l**) and 4T1 cells (**m**) treated with 20 nM bafilomycin A1 (BafA1) for 12 h. Data are representative of at least two independent experiments and are shown as means ± SD.
