# Supplementary material for: Targeting ATAD3A-PINK1-mitophagy axis overcomes chemoimmunotherapy resistance by redirecting PD-L1 to mitochondria
Source: Cell Res. 2023 Jan 10;33(3):215–28. doi: 10.1038/s41422-022-00766-z (PMC9977947; doi:10.1038/s41422-022-00766-z)
Supplement: Supplementary file 5 — Fig. S5 [file 41422_2022_766_MOESM5_ESM.pdf]

Fig. S5

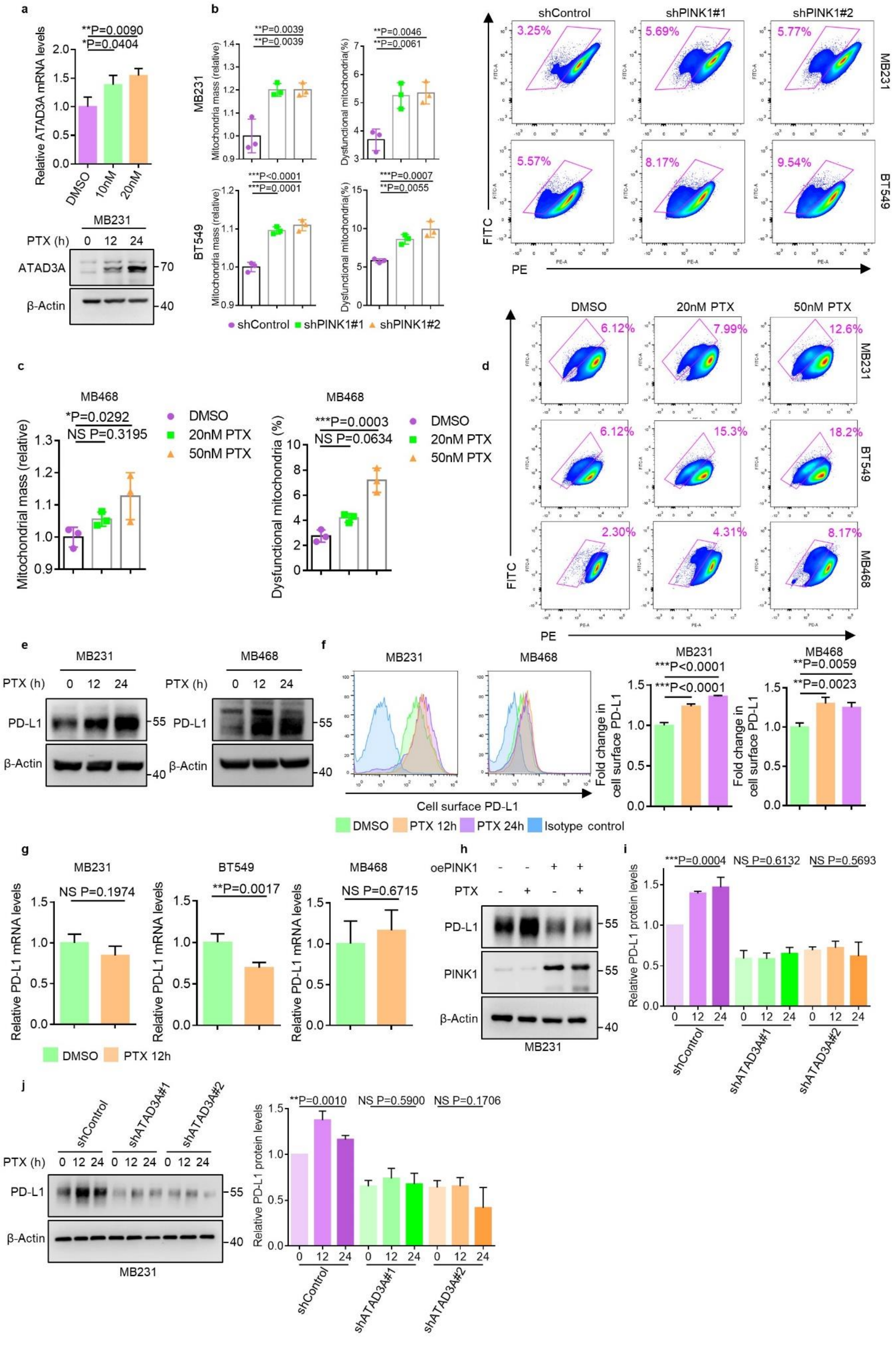
a
Relative ATAD3A mRNA levels
**P=0.0090
*P=0.0404
DMSO
10nM
20nM
MB231
PTX (h)
0
12
24
ATAD3A
β-Actin
70
40
b
MB231
BT549
Mitochondria mass (relative)
Dysfunctional mitochondria(%)
**P=0.0039
**P=0.0039
**P=0.0046
**P=0.0061
***P<0.0001
***P=0.0001
***P=0.0007
**P=0.0055
shControl
shPINK1#1
shPINK1#2
3.25%
5.69%
5.77%
5.57%
8.17%
9.54%
FITC
PE
c
MB468
Mitochondrial mass (relative)
*P=0.0292
NS P=0.3195
Dysfunctional mitochondria (%)
***P=0.0003
NS P=0.0634
DMSO
20nM PTX
50nM PTX
d
DMSO
20nM PTX
50nM PTX
6.12%
7.99%
12.6%
6.12%
15.3%
18.2%
2.30%
4.31%
8.17%
MB231
BT549
MB468
e
MB231
MB468
PTX (h)
0
12
24
PD-L1
β-Actin
55
40
f
MB231
MB468
Cell surface PD-L1
Fold change in cell surface PD-L1
***P<0.0001
***P<0.0001
**P=0.0059
**P=0.0023
DMSO
PTX 12h
PTX 24h
Isotype control
g
MB231
BT549
MB468
Relative PD-L1 mRNA levels
NS P=0.1974
**P=0.0017
NS P=0.6715
DMSO
PTX 12h
h
oePINK1
PTX
PD-L1
PINK1
β-Actin
55
55
40
MB231
i
Relative PD-L1 protein levels
***P=0.0004
NS P=0.6132
NS P=0.5693
0
12
24
shControl
shATAD3A#1
shATAD3A#2
j
shControl
shATAD3A#1
shATAD3A#2
PTX (h)
0
12
24
PD-L1
β-Actin
55
40
MB231
Relative PD-L1 protein levels
**P=0.0010
NS P=0.5900
NS P=0.1706

Fig. S5 Paclitaxel increases PD-L1 accumulation by suppressing mitophagy.

a Top, qRT-PCR of ATAD3A mRNA in MDA-MB-231 cells in the presence of paclitaxel (10 nM or 20 nM) for 12 h (n = 3, one-way ANOVA). Bottom, immunoblot of ATAD3A in MDA-MB-231 cells incubated with 20 nM paclitaxel for 12 h and 24 h. **b** Left, quantification of mitochondrial mass and the percentage of dysfunctional mitochondria in control and PINK1-knockdown cells (n = 3, one-way ANOVA). Right, flow cytometry of mitochondrial mass (vertical axis, assessed with the green fluorescent mitochondrial stain MitoTracker Green FM) and mitochondrial membrane potential (horizontal axis, assessed with the red fluorescent dye MitoTracker Red CMXRos sequestered by active mitochondria). Outlined area, dysfunctional mitochondria. **c** Quantification of mitochondrial mass and the percentage of dysfunctional mitochondria in MDA-MB-468 cells in the presence of paclitaxel (20 nM or 50 nM) for 16 h (n = 3, one-way ANOVA). **d** Flow cytometry of mitochondrial mass and mitochondrial membrane potential. Outlined area, dysfunctional mitochondria. **e** Immunoblot of PD-L1 in MDA-MB-231 cells and MDA-MB-468 cells with 20 nM paclitaxel treatment for indicated times. h, hours. **f** Flow cytometry (left) and quantification (right) of surface PD-L1 in human TNBC cells treated with 20 nM paclitaxel for indicated times. h, hours (n = 3, one-way ANOVA). **g** qRT-PCR of PD-L1 mRNA in human TNBC cells treated with 20 nM paclitaxel for 12 h (n = 3, t-test). **h** Immunoblot of PD-L1 in control and PINK1-overexpressing MDA-MB-231 cells treated with or without 20 nM paclitaxel for 24 h. **i** Quantification of relative PD-L1 protein level in immunoblot, related to Fig. 4k. **j** Left, immunoblot of PD-L1

in control and ATAD3A-knockdown MDA-MB-231 cells treated with or without 20 nM paclitaxel for indicated times. h, hours. Right, quantification of relative PD-L1 protein level in immunoblot. Data are representative of at least three independent experiments and are shown as means ± SD.
