# Supplementary material for: Targeting ATAD3A-PINK1-mitophagy axis overcomes chemoimmunotherapy resistance by redirecting PD-L1 to mitochondria
Source: Cell Res. 2023 Jan 10;33(3):215–28. doi: 10.1038/s41422-022-00766-z (PMC9977947; doi:10.1038/s41422-022-00766-z)
Supplement: Supplementary file 6 — Fig. S6 [file 41422_2022_766_MOESM6_ESM.pdf]

Fig. S6

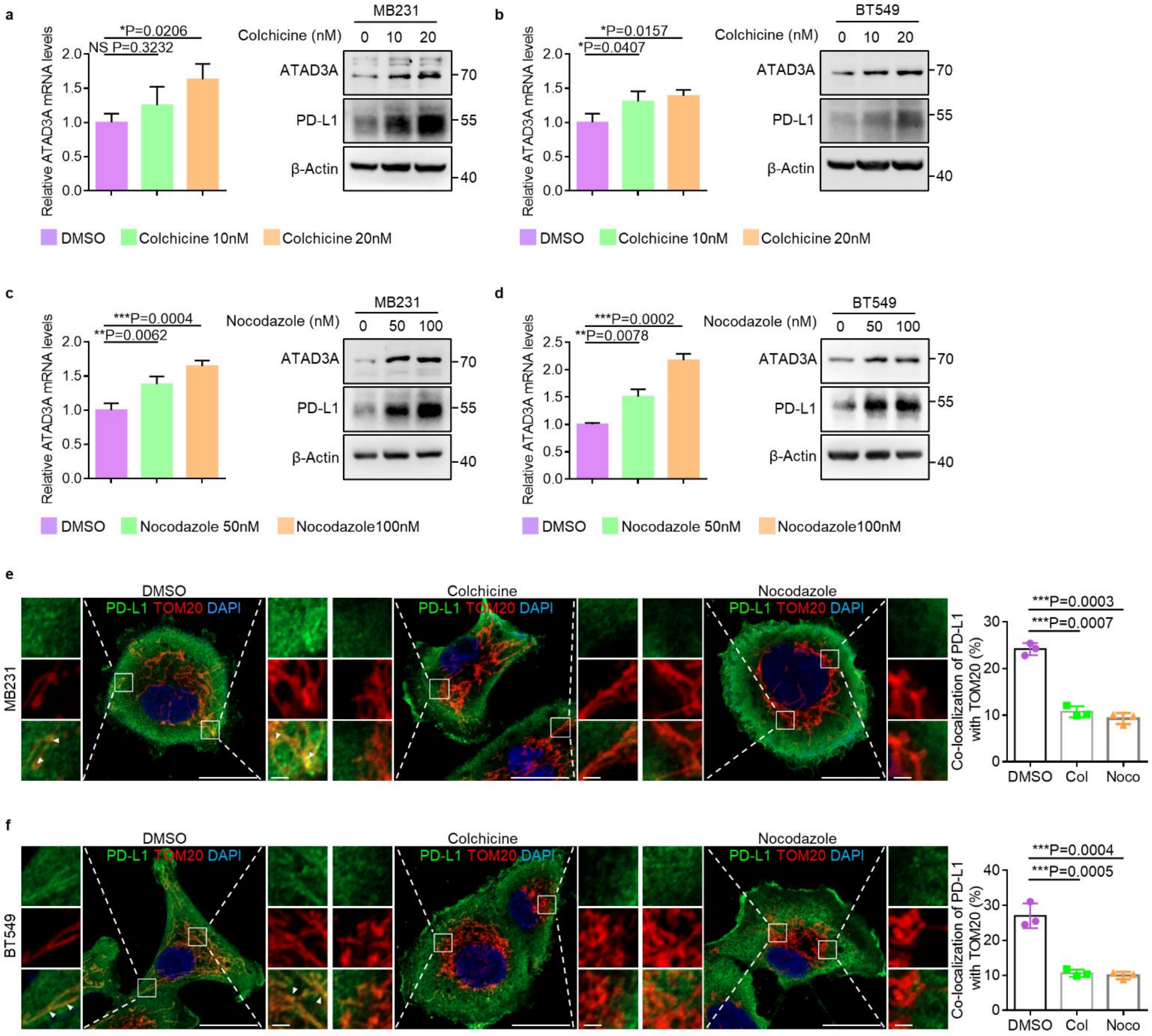

Fig. S6 Colchicine and nocodazole upregulate ATAD3A to reduce the mitochondrial localization of PD-L1.

a, **b** Left, qRT-PCR of ATAD3A mRNA in MDA-MB-231 cells (**a**) and BT549 cells (**b**) in the presence of colchicine (10 nM or 20 nM) for 24 h (*n* = 3, one-way ANOVA). Right, immunoblot of ATAD3A and PD-L1 in MDA-MB-231 cells (**a**) and BT549 cells (**b**) incubated with colchicine for 24 h. **c**, **d** Left, qRT-PCR of ATAD3A

mRNA in MDA-MB-231 cells (**c**) and BT549 cells (**d**) in the presence of nocodazole (50 nM or 100 nM) for 24 h. Right, immunoblot of ATAD3A and PD-L1 in MDA-MB-231 cells (**c**) and BT549 cells (**d**) incubated with nocodazole for 24 h. **e**, **f** Left, co-localization of PD-L1 (green) and TOM20 (red) with or without colchicine (10 nM), nocodazole (100 nM) treatment for 24 h in MDA-MB-231 cells (**e**) and BT549 cells (**f**). Arrowheads, co-localization. Scale bars, 20 μm and 2 μm (inset). Right, the percentage of PD-L1 co-localized with TOM20 (n = 3 fields, t-test). Data are representative of at least two independent experiments and are shown as means ±SD.
