# Supplementary material for: Targeting ATAD3A-PINK1-mitophagy axis overcomes chemoimmunotherapy resistance by redirecting PD-L1 to mitochondria
Source: Cell Res. 2023 Jan 10;33(3):215–28. doi: 10.1038/s41422-022-00766-z (PMC9977947; doi:10.1038/s41422-022-00766-z)
Supplement: Supplementary file 7 — Fig. S7 [file 41422_2022_766_MOESM7_ESM.pdf]

Fig. S7

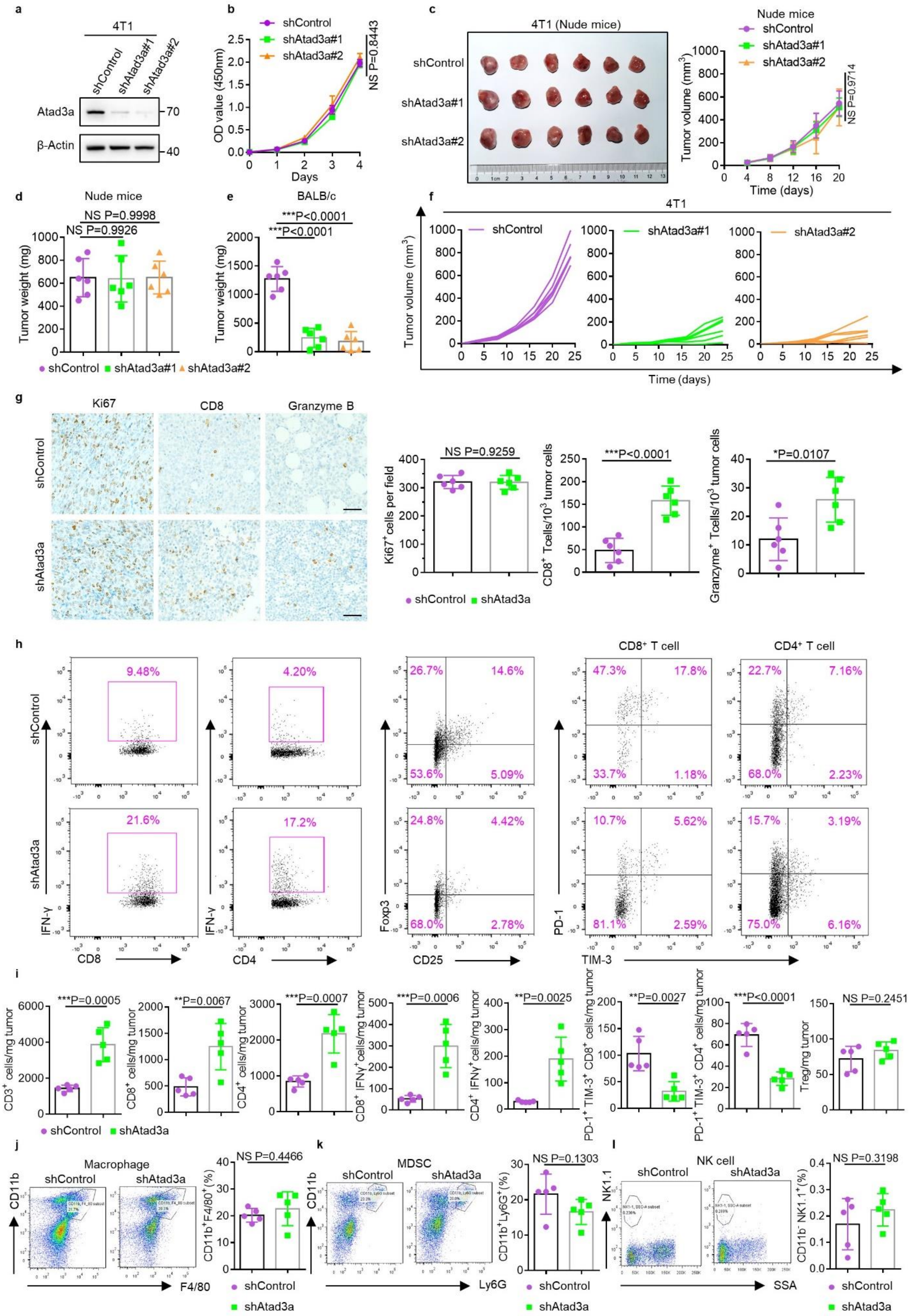

a
4T1
shControl
shAtad3a#1
shAtad3a#2
Atad3a
β-Actin
70
40
b
shControl
shAtad3a#1
shAtad3a#2
NS P=0.8443
OD value (450nm)
Days
c
4T1 (Nude mice)
Nude mice
Tumor volume (mm³)
NS P=0.9714
Time (days)
d
Nude mice
NS P=0.9998
NS P=0.9926
Tumor weight (mg)
e
BALB/c
***P<0.0001
***P<0.0001
f
4T1
Time (days)
g
Ki67
CD8
Granzyme B
shControl
shAtad3a
NS P=0.9259
***P<0.0001
*P=0.0107
Ki67⁺ cells per field
CD8⁺ Tcells/10³ tumor cells
Granzyme⁺ Tcells/10³ tumor cells
h
CD8⁺ T cell
CD4⁺ T cell
IFN-γ
CD8
CD4
Foxp3
CD25
PD-1
TIM-3
i
***P=0.0005
**P=0.0067
***P=0.0007
***P=0.0006
**P=0.0025
**P=0.0027
***P<0.0001
NS P=0.2451
CD3⁺ cells/mg tumor
CD8⁺ cells/mg tumor
CD4⁺ cells/mg tumor
CD8⁺ IFNγ⁺cells/mg tumor
CD4⁺ IFNγ⁺cells/mg tumor
PD-1⁺ TIM-3⁺ CD8⁺ cells/mg tumor
PD-1⁺ TIM-3⁺ CD4⁺ cells/mg tumor
Treg/mg tumor
j
Macrophage
CD11b
F4/80
NS P=0.4466
CD11b⁺F4/80⁺(%)
k
MDSC
Ly6G
NS P=0.1303
CD11b⁺Ly6G⁺(%)
l
NK cell
NK1.1
SSA
NS P=0.3198
CD11b⁻ NK1.1⁺(%)
shControl
shAtad3a

Fig. S7 Depletion of Atad3a in tumor cells suppresses tumor growth in vivo by facilitating anti-tumor immune response.

a 4T1 mouse breast cancer cells were transfected with control shRNA (shControl) or shRNA for Atad3a (shAtad3a#1 and shAtad3a#2). Immunoblot of Atad3a in 4T1 cells. **b** Proliferation of control and Atad3a-knockdown 4T1 cells measured by CCK-8 (Cell Counting Kit-8) assay (n = 5, one-way ANOVA). **c** BALB/c nude mice were inoculated orthotopically with 1×10^5 4T1 cells transfected with control shRNA (shControl) or shRNA for Atad3a (shAtad3a#1 and shAtad3a#2). The endpoint images (left) and volume (right) of tumors formed by control and Atad3a-knockdown cells in BALB/c nude mice (n = 6, one-way ANOVA). **d** The weight of 4T1 tumors formed by control and Atad3a-knockdown cells in nude mice at the end point (n = 6, one-way ANOVA). **e**–**l** BALB/c mice were inoculated orthotopically with control or Atad3a-knockdown 4T1 tumor cells. **e**, **f** The weight at the end point (**e**) and growth curves (**f**) of tumors formed by control and Atad3a-knockdown cells (n = 6, one-way ANOVA). **g** Left, IHC staining of Ki67, CD8 and granzyme B on serial sections of the tumors formed by control and Atad3a-knockdown cells. Right, quantification of the numbers of $Ki67^+$ tumor cells, tumor-infiltrating $CD8^+$ T cells and granzyme B secreting cells (n = 6, t-test). **h** Flow cytometry of tumor-infiltrating $IFN\gamma^+CD8^+$, $IFN\gamma^+CD4^+$, $CD4^+CD25^+Foxp3^+$, $PD\text{-}1^+TIM\text{-}3^+CD8^+$ and $PD\text{-}1^+TIM\text{-}3^+CD4^+$ T cells in tumors formed by control and Atad3a-knockdown 4T1 cells. **i** Quantification of the numbers of tumor-infiltrating $CD3^+$, $CD8^+$, $CD4^+$, $IFN\gamma^+CD8^+$, $IFN\gamma^+CD4^+$, $PD\text{-}1^+TIM\text{-}3^+CD8^+$, $PD\text{-}1^+TIM\text{-}3^+CD4^+$ T cells and $CD4^+CD25^+Foxp3^+$ T_{reg} cells per

milligram of tumor tissue in control and Atad3a-knockdown 4T1 tumors by flow cytometry (*n* = 5, *t*-test). **j–l** Flow cytometry (left) and quantification (right) of the percentages of tumor-infiltrating macrophages (**j**), myeloid-derived suppressor cells (MDSCs) (**k**) and natural killer cells (NKs) (**l**) in 4T1 tumors formed by control and Atad3a-knockdown cells in BALB/c mice (*n* = 5, *t*-test). Data are representative of two independent experiments and were shown as means ± SD.
