# Supplementary material for: Targeting ATAD3A-PINK1-mitophagy axis overcomes chemoimmunotherapy resistance by redirecting PD-L1 to mitochondria
Source: Cell Res. 2023 Jan 10;33(3):215–28. doi: 10.1038/s41422-022-00766-z (PMC9977947; doi:10.1038/s41422-022-00766-z)
Supplement: Supplementary file 8 — Fig. S8 [file 41422_2022_766_MOESM8_ESM.pdf]

Fig. S8

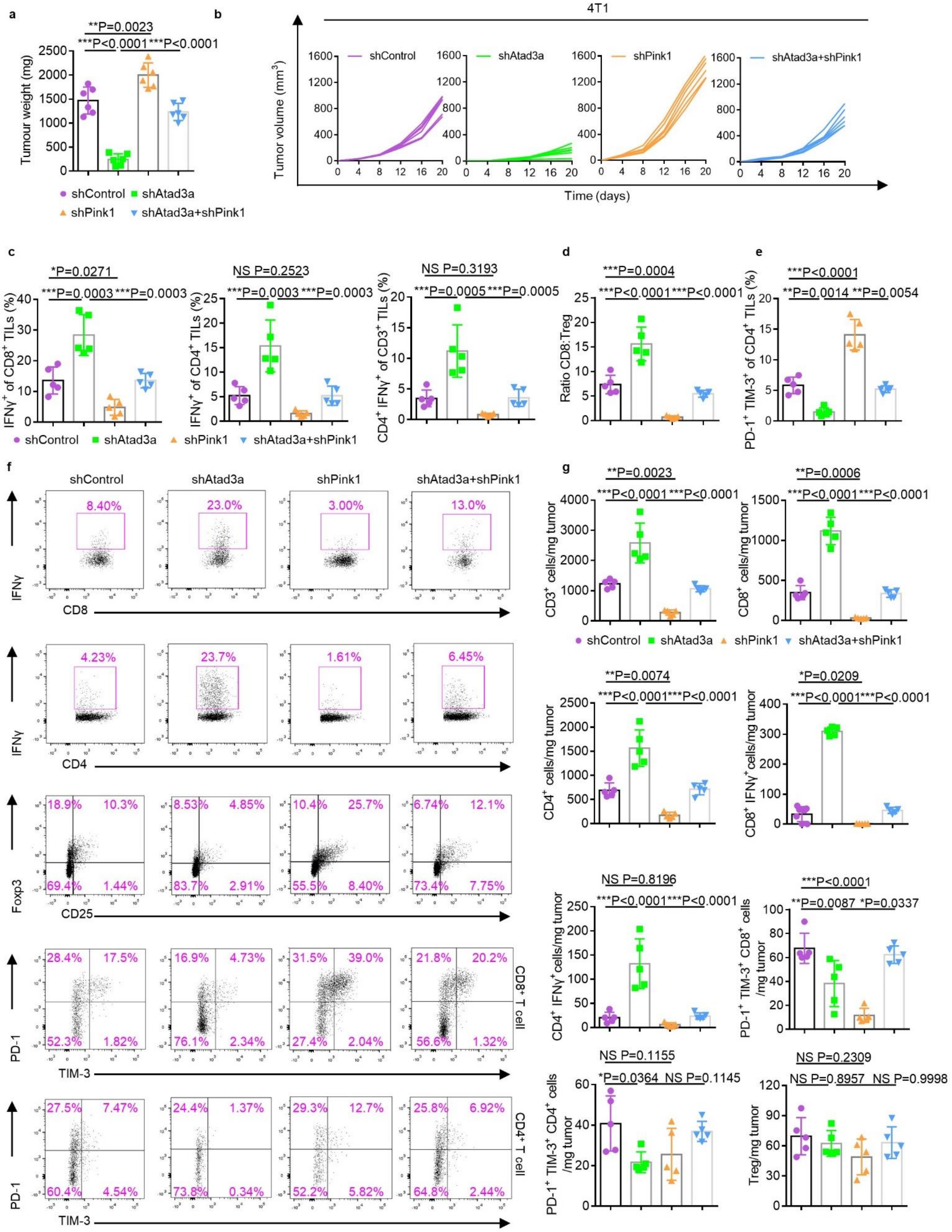

a
b
4T1
shControl
shAtad3a
shPink1
shAtad3a+shPink1
Tumour weight (mg)
Tumor volume (mm3)
Time (days)
**P=0.0023
***P<0.0001 ***P<0.0001
c
d
e
f
g
IFNγ+ of CD8+ TILs (%)
IFNγ+ of CD4+ TILs (%)
CD4+ IFNγ+ of CD3+ TILs (%)
Ratio CD8:Treg
PD-1+ TIM-3+ of CD4+ TILs (%)
*P=0.0271
***P=0.0003 ***P=0.0003
NS P=0.2523
NS P=0.3193
***P=0.0005 ***P=0.0005
***P=0.0004
***P<0.0001 ***P<0.0001
***P<0.0001
**P=0.0014 **P=0.0054
IFNγ
CD8
CD4
Foxp3
CD25
PD-1
TIM-3
CD8+ T cell
CD4+ T cell
CD3+ cells/mg tumor
CD8+ cells/mg tumor
CD4+ cells/mg tumor
CD8+ IFNγ+cells/mg tumor
CD4+ IFNγ+cells/mg tumor
PD-1+ TIM-3+ CD8+ cells /mg tumor
PD-1+ TIM-3+ CD4+ cells /mg tumor
Treg/mg tumor
**P=0.0006
**P=0.0074
*P=0.0209
NS P=0.8196
**P=0.0087 *P=0.0337
NS P=0.1155
*P=0.0364 NS P=0.1145
NS P=0.2309
NS P=0.8957 NS P=0.9998

Fig. S8 ATAD3A-PINK1 mitophagy axis participates in regulating T-cell anti-tumor immune response in vivo.

a–**d** BALB/c mice were inoculated orthotopically with 4T1 cells with control, Atad3a-knockdown, Pink1-knockdown or Atad3a and Pink1 double knockdown. **a** The weight of 4T1 tumors (n = 6, one-way ANOVA). **b** The growth curves of 4T1 tumors. **c** Quantification of the percentages of tumor-infiltrating IFNγ$^+$CD8$^+$ T cells and IFNγ$^+$CD4$^+$ T cells by flow cytometry (n = 5, one-way ANOVA). **d** Ratio of CD8$^+$ cytotoxic T lymphocytes (CTLs) to CD4$^+$CD25$^+$Foxp3$^+$ T_{reg} cells (n = 5, one-way ANOVA). **e** Quantification of the percentage of PD-1$^+$TIM-3$^+$CD4$^+$ T cells by flow cytometry (n = 5, one-way ANOVA). **f** Flow cytometry of tumor-infiltrating IFNγ$^+$CD8$^+$, IFNγ$^+$CD4$^+$, CD4$^+$CD25$^+$Foxp3$^+$, PD-1$^+$TIM-3$^+$CD8$^+$ and PD-1$^+$TIM-3$^+$CD4$^+$ T cells in tumors formed by control, Atad3a-knockdown, Pink1-knockdown or Atad3a and Pink1 double knockdown cells. **g** Quantification of the numbers of tumor-infiltrating CD3$^+$, CD8$^+$, CD4$^+$, IFNγ$^+$CD8$^+$, IFNγ$^+$CD4$^+$, PD-1$^+$TIM-3$^+$CD8$^+$, PD-1$^+$TIM-3$^+$CD4$^+$ T cells and CD4$^+$CD25$^+$Foxp3$^+$ T_{reg} cells per milligram of tumor tissue by flow cytometry (n = 5, one-way ANOVA). Data are representative of two independent experiments and are shown as means ± SD.
