# Supplementary material for: Targeting ATAD3A-PINK1-mitophagy axis overcomes chemoimmunotherapy resistance by redirecting PD-L1 to mitochondria
Source: Cell Res. 2023 Jan 10;33(3):215–28. doi: 10.1038/s41422-022-00766-z (PMC9977947; doi:10.1038/s41422-022-00766-z)
Supplement: Supplementary file 9 — Fig. S9 [file 41422_2022_766_MOESM9_ESM.pdf]

Fig. S9

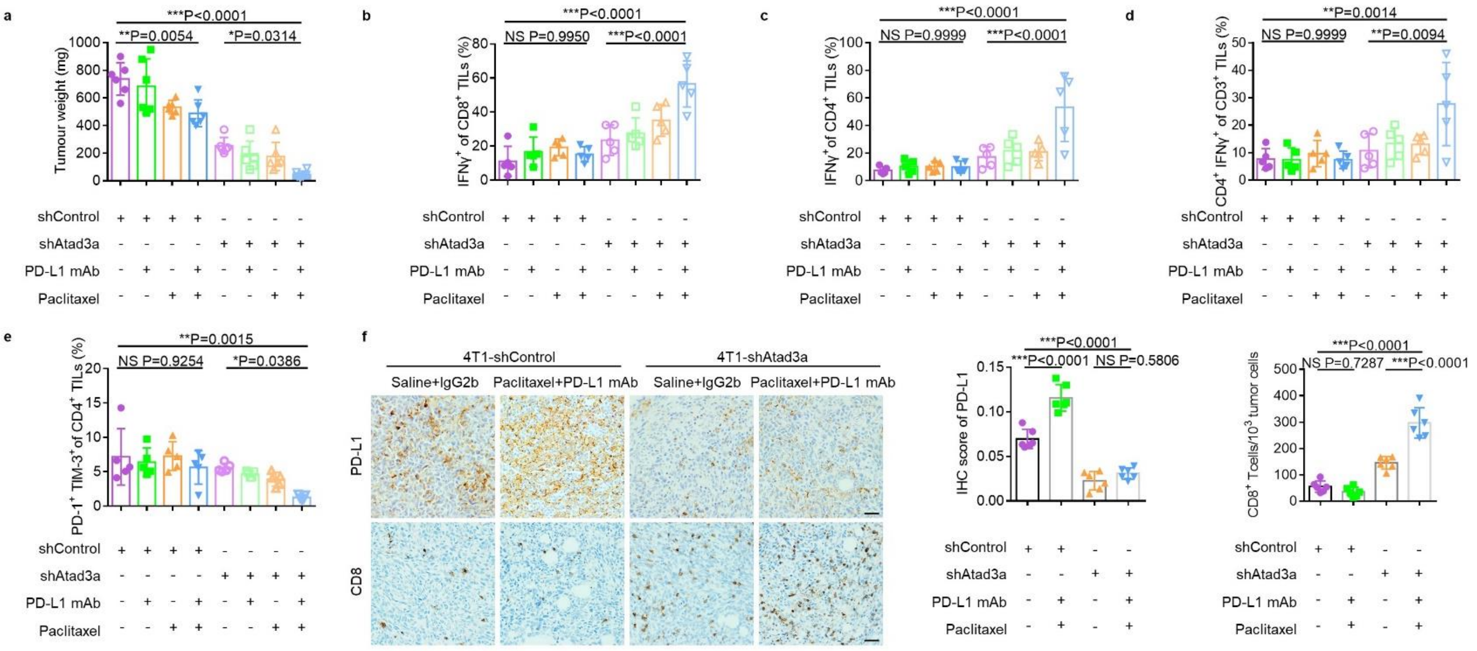

Fig. S9 Knockdown of Atad3a promotes the efficacy of combined therapy with anti-PD-L1 antibody and paclitaxel.

a Tumor volume of orthotopic 4T1 tumors formed by control and Atad3a-knockdown cells with vehicle, anti-PD-L1 monoclonal antibody (PD-L1 mAb), paclitaxel (PTX) or combined anti-PD-L1 antibody with paclitaxel treatment (PD-L1 mAb + PTX). IgG2b and saline were used as controls (n = 6, one-way ANOVA). **b**–**e** Quantification of the percentages of tumor-infiltrating IFNγ^{+}CD8^{+} (**b**), IFNγ^{+}CD4^{+} (**c**, **d**) and PD-1^{+}TIM-3^{+}CD4^{+} T cells (**e**) in 4T1 tumors formed by control and Atad3a-knockdown cells receiving various treatments (n = 5, one-way ANOVA). **f** Left, IHC staining of PD-L1 and CD8 on serial sections of control and Atad3a-knockdown tumors receiving vehicle or anti-PD-L1 antibody plus paclitaxel combination therapy. Scale bars, 50 μm. Right, IHC score of PD-L1 and the quantification of the number of tumor-infiltrating CD8^{+} T cells in control or Atad3a-knockdown tumors with vehicle and combination therapy (n = 6, one-way ANOVA). Data are representative of two independent experiments and are shown as means ± SD.
