# Supplementary material for: Targeting ATAD3A-PINK1-mitophagy axis overcomes chemoimmunotherapy resistance by redirecting PD-L1 to mitochondria
Source: Cell Res. 2023 Jan 10;33(3):215–28. doi: 10.1038/s41422-022-00766-z (PMC9977947; doi:10.1038/s41422-022-00766-z)
Supplement: Supplementary file 10 — Fig. S10 [file 41422_2022_766_MOESM10_ESM.pdf]

Fig. S10

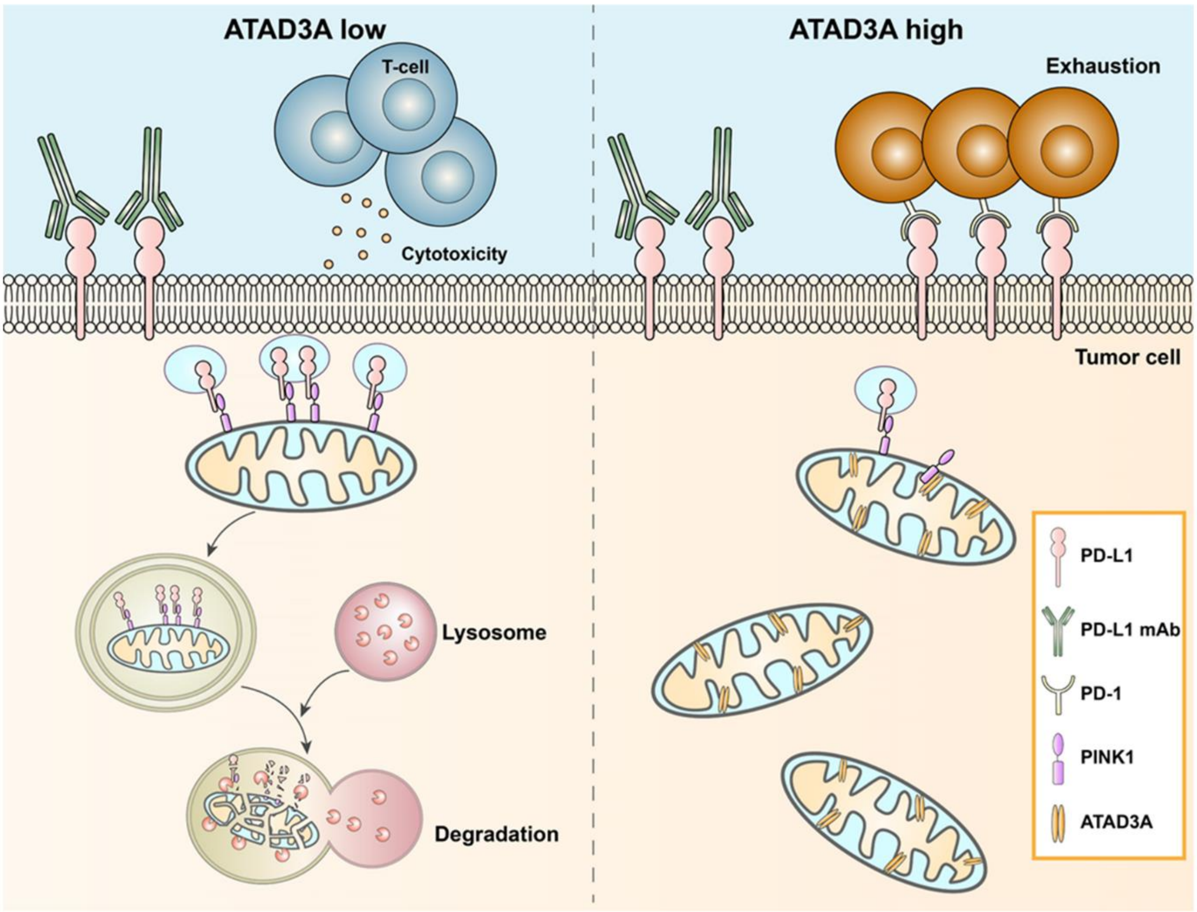

Fig. S10 Diagram illustrating ATAD3A-PINK1-mitophagy regulating the distribution and degradation of PD-L1 in mitochondria.

Schematic model. Left, PD-L1 is recruited to mitochondria by PINK1 and degraded via PINK1-dependent mitophagy in ATAD3A-low tumor cells. PD-L1 remained on cell membrane is neutralized by anti-PD-L1 antibodies, while T cells display cytotoxicity for killing. Right, PINK1-dependent mitophagy is suppressed and PD-L1 is accumulated inordinately on cell membrane to exceed a certain amount of anti-PD-L1 antibodies in ATAD3A-high tumor cells. Excessive PD-L1 on cell membrane binds to PD-1 to inhibit T cell activation.
