# Supplementary material for: Targeting ATAD3A-PINK1-mitophagy axis overcomes chemoimmunotherapy resistance by redirecting PD-L1 to mitochondria
Source: Cell Res. 2023 Jan 10;33(3):215–28. doi: 10.1038/s41422-022-00766-z (PMC9977947; doi:10.1038/s41422-022-00766-z)
Supplement: Supplementary file 11 — Supplementary video legend [file 41422_2022_766_MOESM11_ESM.docx]

**Supplementary information, Videos 1**–**4.**

Video S1, immunostaining of PD-L1 (green) and TOM20-labeled mitochondria (red) with DMSO treatment for 12 h in MDA-MB-231 cells.

Video S2, immunostaining of PD-L1 (green) and TOM20-labeled mitochondria (red) with 20 nM paclitaxel treatment for 12 h in MDA-MB-231 cells.

Video S3, immunostaining of PD-L1 (green) and TOM20-labeled mitochondria (red) with DMSO treatment for 12 h in BT549 cells.

Video S4, immunostaining of PD-L1 (green) and TOM20-labeled mitochondria (red) with 20 nM paclitaxel treatment for 12 h in BT549 cells.
